# An end-to-end deep learning image compression method for satellite images based on entropy model

**DOI:** 10.1371/journal.pone.0355234

**Published:** 2026-08-03

**Authors:** Yanlong Gao, Haiming Xu, Wei Huang, Hao Bai, Boer Peng, Heyang Xu

**Affiliations:** 1 Sichuan Expressway Construction and Development Group Co., Ltd, Chengdu, Sichuan, China; 2 Sichuan Panyan Expressway Construction and Development Co., Ltd, Chengdu, Sichuan, China; 3 School of Information Engineering, Yancheng Institute of Technology, Yancheng, Jiangsu, China; Lincoln University College, MALAYSIA

## Abstract

With the extensive applications of satellite image data in environmental monitoring and geographic surveying and mapping, the amount of data has increased rapidly, which brings great challenges for transmitting and storing these images. However, when processing high-resolution and multi-spectral satellite data, existing image compression methods often lead to low compression efficiency or poor reconstruction image quality due to its insufficient generalization ability. In this paper, we propose an end-to-end deep learning image compression framework for visible infrared imaging radiometer suite (VIIRS) satellite imagery. The framework consists of an analysis transform encoder, a synthesis transform decoder, a hybrid training-testing quantizer and a probability model for entropy coding. A cumulative distribution function (CDF) is constructed to compute the discrete likelihood of quantized latent symbols under a Gaussian-mixture entropy model. It is integrated with a checkerboard context structure and a VIIRS-oriented block-processing pipeline. Finally, we conduct systematic experiments based on NASA VIIRS multi-spectral datasets. Experimental results show that the proposed method achieves 0.51 ± 0.04 bpp, 38.39 ± 0.72 dB PSNR and 0.973 ± 0.007 SSIM. Relative to ELIC and the Transformer-CNN baseline, it reduced bpp by 13.6% and 10.5% and improved PSNR by 0.78 dB and 0.61 dB, respectively. The framework can compress the data volume to approximately 1.5%−4% of the original size, corresponding to an average compression ratio of about 30:1. In order to meet the processing requirements of high-resolution satellite images, we further propose a block compression strategy, which divides large-size images into sub-blocks of 256 × 256 pixels for independent compression, and realizes complete image reconstruction through decompression and splicing technology.

## 1. Introduction

As an important product of modern remote sensing technology, satellite image data has become a core supporting tool in agricultural monitoring, urban planning, disaster early warning and other fields [1]. Taking the meteorological satellite system as an example, it can realize all-weather and multi-dimensional monitoring of the earth’s surface and atmosphere by carrying a variety of optical remote sensing sensors (including panchromatic cameras, multi-spectral and hyper-spectral imaging equipment), covering a wide spectrum of electromagnetic wave information from ultraviolet to infrared. These high-precision remote sensing data play an irreplaceable role in key fields such as natural phenomenon monitoring, weather system evolution analysis, and ecological environment assessment [2]. With the rapid development of remote sensing technology, the amount of satellite data has shown an exponential growth trend. Taking China’s Fengyun series of meteorological satellites as an example, the total amount of multi-spectral data generated daily has exceeded 500 TB, which is widely used in major scientific research projects such as typhoon track prediction and climate model construction [3]. According to the latest research data, a single remote sensing satellite can transmit up to 1.2TB of data per orbit, and the total amount of image data transmitted by the ISS per day is equivalent to the storage capacity of 300 4K high-definition movies [4]. The explosive growth of data on this scale poses unprecedented challenges to data transmission efficiency and storage system capacity, and has become a bottleneck restricting the application of remote sensing technology.

With the explosive growth of remote sensing satellite images, traditional data compression technology is facing severe efficiency and accuracy challenges. First, the complexity of the compression process is dramatically amplified on large data sets. Traditional methods often rely on a series of complex pre-processing steps, including geometric correction and radiometric calibration [5]. These steps can ensure the accuracy of small-scale data, but when processing data streams such as 500 terabytes per day, the huge time and computing resource consumption have become unbearable. For example, when the wavelet transform is used to compress sea temperature data in marine monitoring, it is found that when the compression ratio is set to 8:1, the mission-critical accuracy of front detection is significantly reduced by about 29% [6]. Second, the empirical characteristics of traditional methods and the limited generalization ability constitute another major bottleneck. Its core algorithms are mostly based on manual design based on domain-specific knowledge, which not only requires deep expert intervention, but also leads to insufficient adaptability of the model in the face of diverse feature scenarios. Relevant studies show that although the application of JPEG2000 standards to compress satellite images can achieve about 1/8 of the volume reduction, it causes up to 17.3% of the loss of feature information in quantitative analysis applications, such as vegetation coverage [7]. More importantly, traditional compression strategies are fundamentally flawed because they cannot adapt to the inherent spatial heterogeneity of remote sensing images. For example, the difference in effective information density between urban and forest areas can be as high as 5.6 times in the same satellite image [8]. However, the unified compression ratio used by traditional methods cannot dynamically adjust the information density in different regions, resulting in uneven spatial preservation efficiency of information and further weakening the utilization value of data. Therefore, exploring a new generation of compression paradigms that can adapt to spatial changes has become an urgent need to break through the current bottleneck of satellite data processing.

In order to cope with the above challenges, satellite big data intelligent compression technologies with artificial intelligence come into being naturally [9–15]. They aim to use intelligent algorithms and advanced computing models to efficiently process and compress large-scale image data obtained from satellite sensors. On the one hand, relying on the support of advanced computing power such as graphics processing units (GPUs), the traditional complex multi-step processing process can be integrated into an end-to-end artificial intelligence compression framework, thereby improving the processing efficiency and compression performance of massive satellite images. On the other hand, by training on massive historical satellite datasets, the model can independently mine its internal statistical laws and structural features according to inputs and annotations, reducing the dependence on manual feature design and domain expert knowledge. Because of the strong nonlinear characterization ability of the deep learning model, it shows strong generalization ability in the face of diverse feature scenarios, ensuring the accuracy of compressed reconstruction results and providing a solid data foundation for downstream quantitative remote sensing analysis tasks.

In recent years, satellite image data compression technology based on artificial intelligence has attracted widespread attentions [16–28]. With the breakthrough progress of deep learning and neural networks in computer vision, natural language processing and other fields, their migration and application to remote sensing image compression have shown significant technical potential. However, the direct application of existing general-purpose deep learning models to satellite image compression still faces key technical bottlenecks and adaptability challenges. The results show that significant geometric distortions are induced when compression is performed using a general object detection model based on natural image training. For example, an average of 3.2 pixels of pixel displacement is generated at the edge of a building, which directly affects the geometric accuracy of the data and the credibility of subsequent analysis [21]. The root cause of this phenomenon is that the traditional entropy model is difficult to accurately fit the highly non-stationary and alienated pixel value probability distribution characteristics in remote sensing images. For example, aurora observation data shows that the probability density of electromagnetic radiation intensity in a specific interval can reach 7.8 times that of ordinary natural scenes [22], which poses a serious challenge to traditional models.

It is worth noting that existing studies have made progress by introducing more flexible entropy models. For example, in the compression of marine monitoring data, the hybrid entropy model successfully improved the signal-to-noise ratio by 2.7 dB [23]. This also verifies the feasibility of improving the performance path by optimizing probabilistic modeling from the practical level. Therefore, this paper proposes an end-to-end deep learning image compression framework based on the Cumulative Distribution Function (CDF) to calculate the probability value of entropy models, aiming to further improve the compression performance and reconstruction quality of satellite images. The summary of our contributions are as follows.

First, we proposed an entropy-model component, which is a CDF-guided implementation of discretized Gaussian-mixture likelihood estimation for quantized latent symbols. The novelty lies in adapting this likelihood calculation to VIIRS multispectral blocks and coupling it with a checkerboard context structure that supports parallel probability estimation.

Second, we construct an end-to-end satellite-image compression framework, which includes an encoder, a decoder, a quantizer, hyperprior/context modules and entropy coding. The framework integrates residual bottleneck blocks and simplified attention modules to balance reconstruction quality, bitrate and computational cost.

Third, systematic experiments based on NASA VIIRS multi-spectral datasets are conducted to verify the validity of the proposed method. The results show that our study can compress the data volume to a interval of 1.5%−4% compared with original size and the average compression ratio reaches about 30:1.

Finally, a block processing scheme suitable for large-scale satellite images is proposed, which is based on the segmentation and compression strategy of 256 × 256 pixel sub-blocks. This scheme combines with decompression and splicing technology to achieve large-size image processing, which effectively reduces the memory requirement through parallel computing and thus enables the system to adapt to practical processing scenarios of tens of GB of satellite data.

Although several used blocks, such as scale hyperpriors, autoregressive context models, attention modules and Gaussian-mixture likelihoods are inherited from the learned-compression literature,the specific contribution of this work is their integration and adaptation to VIIRS satellite-image compression, including CDF-based discrete likelihood calculation over quantization bins, checkerboard mask-kernel context implementation, HDF5 multispectral preprocessing, block-wise compression and reconstruction, and an expanded satellite-oriented evaluation protocol. The detailed novelty comparison between the proposed method and representative learned image-compression methods are listed in Table 1.

**Table 1:** Novelty comparison between the proposed method and representative prior learned image-compression methods.

Methods	Main ideas	Context and computational characteristics	Distinction from the proposed VIIRS framework
Ballé et al. [29]	Hyperprior and Gaussian likelihood.	Efficient parallel coding, but without an explicit spatial context model.	The proposed method adds a CDF-guided Gaussian mixture, checkerboard context, and VIIRS-specific preprocessing and block reconstruction.
Minnen et al.[30]	Hierarchical priors with an autoregressive context model.	Strong spatial modeling, but serial autoregressive decoding limits parallelism.	The proposed method replaces strict serial context prediction with checkerboard parallel estimation for satellite patches.
Cheng et al. [28]	Discretized Gaussian mixture likelihoods and attention modules for natural images.	Accurate entropy modeling for natural images; context evaluation is not tailored to VIIRS data.	The proposed method adapts CDF likelihood calculation, simplified attention, HDF5 band processing, and large-scene block stitching to VIIRS imagery.
ELIC [10]	Uneven channel grouping with spatial-channel contextual adaptive coding.	Strong rate-distortion and runtime performance through channel-wise context modeling.	ELIC is included as a strong benchmark; the proposed method emphasizes checkerboard parallelism and an end-to-end satellite deployment pipeline.
Transformer-CNN compression [35]	Mixed Transformer-CNN blocks for local and global feature modeling.	Powerful global modeling, but with greater architectural and computational complexity.	The proposed framework uses residual bottlenecks and simplified attention together with satellite-specific entropy and block-processing modules.
Proposed method	Residual encoder-decoder, hybrid quantization, CDF-guided Gaussian mixture, hyperprior, checkerboard context, and block processing.	Parallel context estimation with a compact satellite-oriented architecture.	Integrates the learned components into a reproducible VIIRS workflow and evaluates them through modern baselines and component-wise ablation.

The rest of the paper is organized as follows. Section 2 presents the proposed deep learning image-compression framework. Section 3 describes the entropy model, CDF-based discrete likelihood calculation, arithmetic coding, and context structure. Section 4 reports the dataset preprocessing, experimental setup, evaluation metrics, baseline comparison protocol, ablation design, and result analysis. Finally, we conclude the paper and discuss limitations and future work in Section 5.

## 2. Image Compression Model based on Deep Learning (ICMDL)

**Fig 1:**
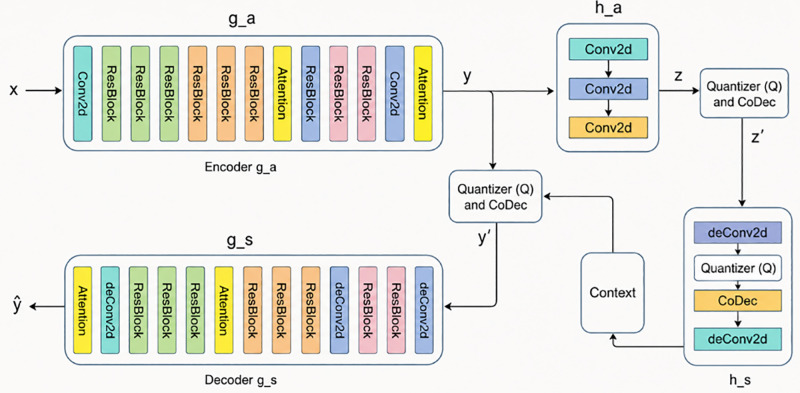
Deep learning-based image compression and decompression framework.

The framework of the proposed ICMDL is shown in Fig 1, which is mainly composed of an analysis transform encoder (g_a), a synthesis transform decoder (g_s), a quantizer, hyperprior transforms (h_a and h_s), a context model and an entropy CoDec. The encoder adopts multi-level convolution and pooling operations to gradually reduce the spatial dimension of the feature map and realize the compressed representation of information. The decoder gradually restores the image resolution through de-convolution and up-sampling operations. The hyperprior and context modules estimate probability parameters for the quantized latent symbols and then arithmetic coding converts the symbols into a compact bitstream; The model adopts an end-to-end training strategy, with mean square error (MSE) as the loss function, and combines the Adam optimizer for parameter optimization.

The image compression process is as follows: Given an input image x, the encoder g_a maps it into a continuous latent representation y = g_a(x). The quantizer Q, which corresponds to the “Q” partof the “Q&CoDec” module in Fig 1, discretizes y into the quantized latent representation, denoted as y’. The entropy coder/decoder then encodes and decodes y’ according to the probability distribution estimated by the entropy model. Finally, the decoder g_s reconstructs the image as $\hat{y}=g\_s(y\text{'})$. Finally, the compressed code stream and the probability distribution features together constitute a complete compressed representation, which not only retains the essential features of the image, but also realizes efficient compression through quantization and probability modeling. This process reflects the synergy between end-to-end optimization and probabilistic priori guidance, which can significantly improve the compression efficiency and reconstruction quality.

In addition to the main encoder-quantizer-decoder pathway, the framework introduces a hyper-prior branch composed of h_a and h_s. The module h_a, called the hyper-analysis transform, takes the latent representation y as input and extracts its higher-order statistical dependency to generate the hyper-latent variable z = h_a(y). Then, z is quantized into z’, and encoded as side information. The module h_s, called the hyper-synthesis transform, decodes z’ to predict the distribution parameters of y’, such as the mean, scale or mixture parameters. These parameters are combined with the context model to estimate p(y’|z’), which provides probability information for arithmetic entropy coding.

### 2.1. Encoder construction

In the image compression framework based on convolutional neural network, the encoder converts the image into a low-dimensional latent representation through nonlinear mapping, which provides feature support for compression and reconstruction. The encoder constructed in this paper fuses the residual bottleneck network, the 1 × 1 convolutional compression layer and the self-attention mechanism to realize multi-scale feature fusion and dynamic weight distribution. Among them, the residual bottleneck network alleviates the disappearance of gradients through cross-layer connections and enhances the ability of deep feature expression. The 1 × 1 convolutional layer compresses the channel dimension to reduce the computational complexity; Through global context modeling, the self-attention network focuses on the key areas in a self-adaptive manner and improves feature discrimination. The structure combines hierarchical feature extraction and dynamic attention mechanism to significantly improve the reconstruction quality while ensuring compression efficiency, which can provide a scalable scheme for deep learning image compression.

As the core module of deep residual network, the residual bottleneck network effectively solves the problems of gradient disappearance and gradient explosion in deep neural network training through innovative structural design. The module adopts the “bottleneck” structure of halving the number of feature channels and then reducing to perform compact feature representation learning in low-dimensional feature space, which significantly improves the efficiency and discriminative performance of feature extraction. When the feature dimension is reduced to the original scale, the refined features can be recombined, thereby enhancing the nonlinear expression ability of the network. This three-stage design of compression-processing-expansion not only reduces computational complexity, but also ensures the stability of gradient propagation through the residual connection mechanism. In the process of image compression, the structure achieves a balance between computational efficiency and feature expression ability through multi-scale feature fusion, providing efficient feature extraction capabilities for encoders. Based on this, this paper utilizes the residual bottleneck network to construct an image compression encoder, which aims to improve the performance of the model on maintaining low computational overhead while obtaining better compression performance.

**Fig 2:**
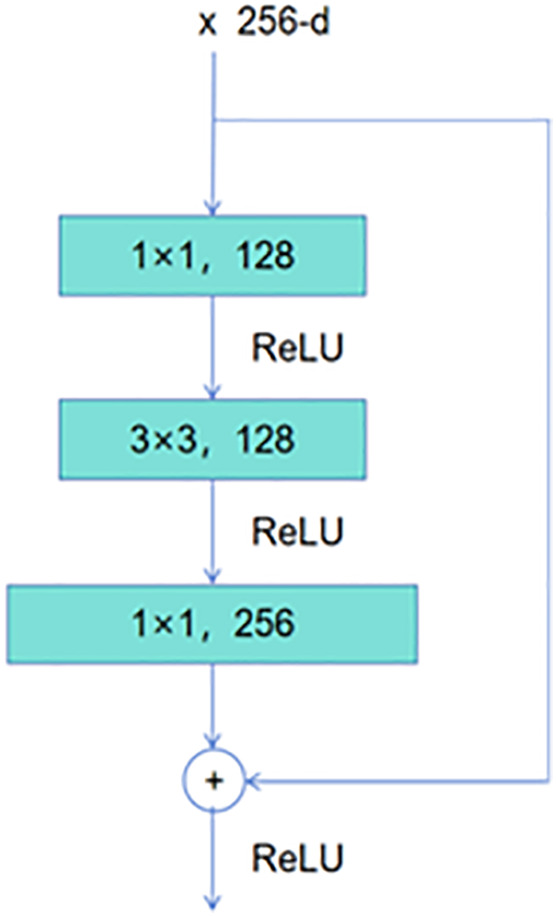
Residual bottleneck network structure.

The traditional residual network usually adopts a double 3 × 3 convolutional structure, which can effectively extract spatial features, but will cause the problem of computational redundancy. This paper proposed an improved residual bottleneck network to realize the dynamic regulation of channel dimension through a 1 × 1 convolution, as shown in Fig 2. The structure adopts a three-stage design of “compression-processing-expansion”. Firstly, the number of input channels is compressed to 1/4 through a 1 × 1 convolution and redundant information is eliminated in low-dimensional space. Then, a 3 × 3 convolution is used to extract key spatial features and the discriminative ability is enhanced through cross-channel interaction. Finally, the number of channels is restored to the original scale through another 1 × 1 convolution and the feature expression ability is reconstructed. The design maintains the advantages of residual connection while reducing the computational cost by 75% through channel compression, so as to achieve a balance between computational efficiency and feature expression ability.

**Fig 3:**
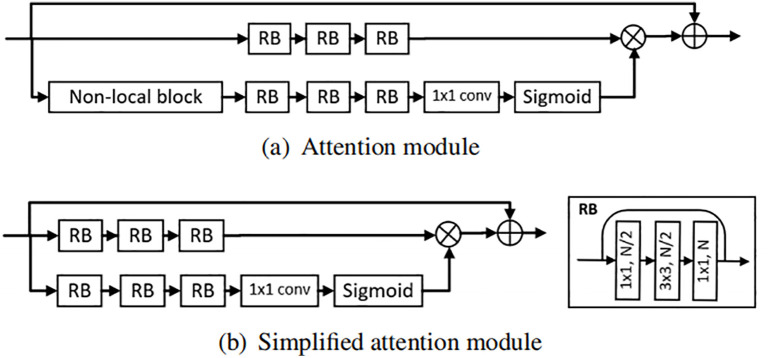
The structure of the attention module.

Through the dual weighting mechanism of channel dimension and spatial position, the attention module realizes the prominent extraction of important feature channels and the precise focus of complex spatial areas. The module adopts the residual connection design to alleviate the problem of gradient disappearance in deep network training, which provides a stable optimization path for feature representation learning. Chen et al. proposed two variants of the attention module in the image compression algorithm, as shown in Fig 3. Among them, the initial version, shown in Fig 3(a), adopts a non-local block structure, which can effectively capture long-distance dependencies, but the computational complexity increases significantly, resulting in a significant decrease in training efficiency. The simplified version, shown in Fig 3(b), retains the core mechanisms of channel attention and spatial attention, reducing training time by 30% by removing non-local blocks. Although the feature represents a slight reduction in performance, it is still significantly better than the baseline model without the attention module. The simplified attention module adopted in this paper contains two key components: 1)The channel attention module realizes the dynamic weighting of feature channels through global average pooling and full connection layer; 2) The spatial attention module completes the weight allocation of spatial position through the fusion operation of channel mean and maximum value.

**Fig 4:**
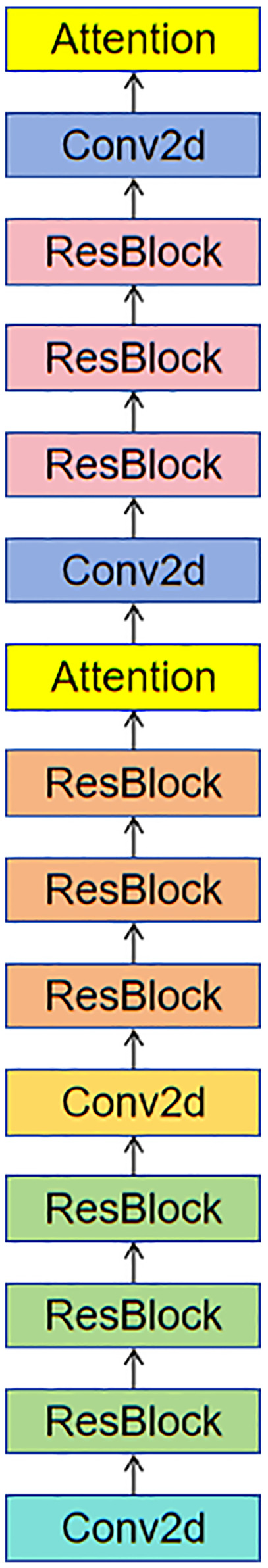
The network structure of the Encoder.

The encoder structure proposed in this paper is shown in Fig 4, in which Conv2d represents the two-dimensional convolutional layer, ResBlock represents the residual bottleneck block and the Attention module is used for the attention mechanism. The encoder is mainly composed of three modules: the first module contains a Conv2d layer and three ResBlocks; The second module contains a Conv2d layer, three ResBlocks and an Attention module; The last module consists of a Conv2d layer, three ResBlocks, one Conv2d layer and an Attention module. The structure realizes feature extraction and information enhancement through residual connection and attention mechanism, which is suitable for image processing tasks.

The design of the encoder maintains the advantages of residual connection while reducing the computational cost by about 75% through lightweight structure, so as to achieve a balance between computational efficiency and feature expression ability, providing an efficient feature enhancement scheme for image compression encoders.

### 2.2. Decoder construction

**Fig 5:**
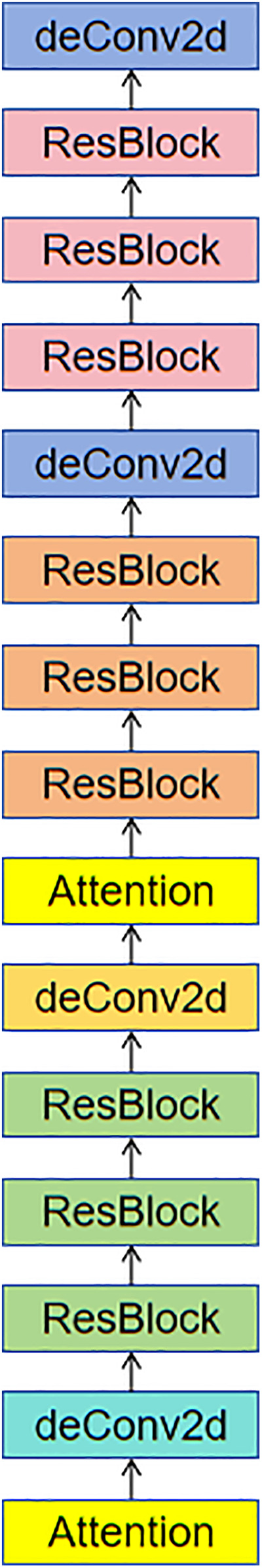
The network structure of the Decoder.

The decoder plays a key role in image generation models and its core function is to reconstruct the quantized latent representation into the original image and strive to make the reconstructed image highly consistent with the original image in terms of visual characteristics. To achieve this goal, the decoder adopts a symmetrical dual structure design with the encoder, which not only maintains the consistency of the model, but also significantly improves the accuracy and detail restoration ability of image reconstruction by introducing a residual bottleneck network and an attention mechanism. The residual bottleneck network effectively alleviates the common gradient disappearance problem in deep neural network training through cross-layer connection, while the attention module enhances the model’s ability to capture key features of images through dynamic weight allocation. In addition, the decoder extensively integrates the residual network (ResNet) structure to further optimize the training stability of the deep network. The decoder structure designed in this paper is shown in Fig 5, in which deConv2d represents two-dimensional deconvolution. The designed decoder consists of three functional modules. The first module contains a Attention, a deConv2d and three ResBlocks. The second module consists of a deConv2d, one Attention and three ResBlocks. The third integrates a deConv2d, one Attention and another deConv2d. This hierarchical modular design can realize accurate mapping from low-dimensional latent space to high-dimensional image space through gradual up-sampling and feature fusion, providing a structural guarantee for high-quality image reconstruction.

### 2.3 Quantizer construction

In the deep learning-based image compression framework, the quantizer is responsible for converting continuous signals (such as the latent representation of convolutional neural network output) into discrete values, and its performance directly affects the image compression efficiency and reconstruction quality. The traditional image compression framework usually adopts the uniform scalar quantization method to achieve discretization through rounding operation, but the gradient is zero in the back-propagation process, resulting in the model being underivable. This feature makes it difficult to directly apply uniform scalar quantization to deep learning frameworks, as it can’t support gradient descent optimization and, in turn, limits the performance improvement of models in end-to-end training. Therefore, it is necessary to explore an alternative quantization strategy to solve the problem of gradient disappearance and ensure the derivability and optimization feasibility of the compression framework.

This paper proposes a hybrid quantization strategy, which combines noise quantization with uniform scalar quantization to solve the gradient vanishing problem in the deep learning image compression framework. In the training phase, the noise quantization method is used to ensure model derivability. During the testing phase, it switches to traditional uniform scalar quantization to maintain efficient compression performance. This strategy effectively balances training optimization and testing efficiency, providing a feasible quantitative solution for deep learning image compression. The noise quantization method simulates the quantization effect by adding uniform noise to the continuous latent representation *y*, as shown in Eq. (1).


$$\begin{array}{c} \hat{y}=y+\varepsilon ,\;\varepsilon \;U(-0.5,\;0.5) \end{array}$$ (1)

The uniform scalar quantification adopts the rounding method, whose calculation is shown in Equation (2). It is used in the latent quantization stage, specifically in both the main latent representation *y* generated by g_a and the hyper-latent representation *z* generated by h_a.


$$\begin{array}{c} \hat{y}=\Delta \cdot round(y/\Delta ) \end{array}$$ (2)

The proposed hybrid quantization strategy combines noise quantization and uniform scalar quantization, which aims to solve the quantization compatibility problem in the training and testing stages of deep learning image compression framework. In the forward propagation process, noise quantization avoids the discretization jump caused by traditional hard quantization by introducing uniformly distributed random noise simulation quantization errors, so that the loss function can maintain gradient continuous transmission during back-propagation, ensuring the conductability of end-to-end training and improving the stability of model convergence. However, the strategy adopts differentiated quantization operations in the training and testing stages, which may cause the error distribution of the model to be inconsistent with the training stage during inference, which in turn leads to a decrease in the quality of reconstruction. Therefore, how to minimize the performance loss caused by training-test quantization differences while maintaining training derivability becomes the key optimization direction of this hybrid quantization strategy.

## 3. Entropy model of end-to-end image compression

The entropy model is an important module in the end-to-end image compression framework, which guides the encoding process by estimating the probability distribution of the potential representation *y* of the compressed image. It constructs a probability distribution of potential representation *y* through a deep neural network to guide the coding process to achieve efficient compression. The model adopts an adaptive probability prediction mechanism to guide the arithmetic encoder to encode the latent representation at the minimum bit rate. At the same time, combined with context-dependent modeling, the adaptability to local features is improved. The model realizes lossless compression of images based on accurate probability estimation through arithmetic coding optimization, which significantly reduces the bit rate. In addition, the entropy model is jointly optimized with analytical transformation and synthetic transformation, and the utilization rate distortion is combined with the loss function for end-to-end training, so as to balance the compression rate and reconstruction quality, and improve the overall compression performance.

### 3.1. Entropy model

**Fig 6:**
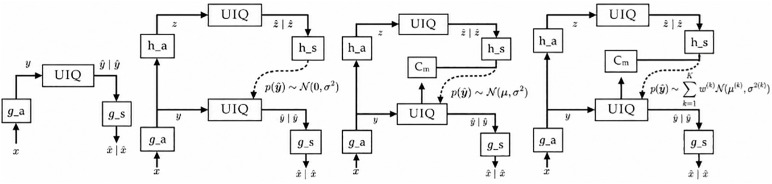
Four structures of entropy model. (a) Baseline (b) Hyperprior (c) Joint (d) Mixture.

In traditional image compression models, entropy encoding usually adopts Huffmann coding or adaptive arithmetic coding methods. These coders are efficient only when the symbol probabilities are accurately estimated. Learned compression methods use neural networks to estimate the probability distribution of quantized latent representations. Therefore, the proposed image compression framework based on deep learning can effectively utilize neural networks to dynamically predict the probability distribution of potential representations, which improves the coding efficiency. Fig 6 shows four typical entropy model structures, i.e., a factorized baseline model, a hyperprior model, a joint autoregressive-hyperprior model, and a Gaussian-mixture model.

Fig 6(a) shows an entropy model of traditional Huffman encoding or adaptive arithmetic encoding, which directly encodes the latent representation of the encoder’s output. This method has limitations in processing different image contents and cannot effectively adapt to the changes in statistical characteristics.

Fig 6(b) is a super-prior model [29], which dynamically controls the probability distribution (denoted by $P\text{\textbackslash{}hspace\{0.17em}\}(\hat{y}|\hat{z})$) of the potential representation $\hat{y}$ by introducing a hidden variable $\hat{z}$. This method hypothesizes that the elements of the latent representation are independent and identically distributed, and obey the Gaussian distribution with a mean of 0 and a variance of *σ*_*i*_. It can more accurately model the spatial dependencies of potential representations and thus improve the compression efficiency.

Fig 6(c) is a joint model [30], in which a contextual model is introduced. The authors expand the latent representation distribution to a Gaussian distribution with a mean of *μ*_*i*_ and a variance of *σ*_*i*_. The model dynamically adjusts the distribution parameters to make the probability estimation of potential representations more refined.

Fig 6(d) shows a hybrid model proposed in [31], which uses the hybrid Gaussian distribution to model the latent representation to achieve more flexible probability estimation. Compared to a single Gaussian distribution, hybrid models perform better in complex images, providing higher compression efficiency and reconstruction quality.

In this paper, the probability of a quantized latent value *k* is obtained by integrating the predicted continuous density over the quantization interval [*k*-0.5, *k* + 0.5]. For a Gaussian component with mean *μ* and standard deviation *σ*, this discrete probability is computed as the CDF difference shown in Equation (3). For a Gaussian-mixture model, the final probability is the weighted sum of the CDF differences over all mixture components.


$$\begin{array}{c} P\left(k\right)=\Phi \left(\frac{k+0.5-\mu }{\sigma }\right)-\Phi \left(\frac{k-0.5-\mu }{\sigma }\right), \end{array}$$ (3)

in which Φ is the standard Gaussian Cumulative Distribution Function(CDF). *μ* and *σ* are the predicted mean and standard deviation, respectively and *k* is the quantized latent symbol.

### 3.2. The coding method of entropy model

The joint entropy model adopts the arithmetic coding, which is an efficient non-destructive entropy coding method and can achieve a compression rate close to the Shannon entropy coding limit. When each element of the symbol sequence {*S*_*i*_} is known and obeys the same probability distribution *P*(*S*_*i*_), the arithmetic coding can realize data representation by mapping the symbolic sequence {*S*_*i*_} to a real number *x*∈[0,1), and its binary form is the compressed code stream. The specific processes are as follows: First step obtains the input symbol *S*_*i*_ and sets the initial coding interval as [0,1]; Second step gets the probability *P*(*S*_*i*_) of the symbol *S*_*i*_; Step 3 calculates the cumulative probability interval of the symbol *S*_*i*_, denoted by [*l*_*i*_, *h*_*i*_]; Step 4 updates the probability interval (denoted by [*L*, *H*]) of the current coding interval of *S*_*i*_ and its upper and lower limits are calculated by Equation (4).


$$\begin{array}{c} \left\{ \begin{array}{c} @c@L\text{'}=L+(H-L)\cdot I_{i}\\ H\text{'}=H+(H-L)\cdot H_{i} \end{array}\right. \end{array}$$ (4)

Repeat steps 3 and 4 above-mentioned to update the mapping interval until the symbol encoding is completed, and the final mapping interval can be obtained. Finally, the shortest binary decimal in the final mapping space is selected as the encoding of the character. The advantage of arithmetic coding lies in its high efficiency and precision, especially when it comes to dealing with continuous probability distributions.

### 3.3. The context structure of entropy model

**Fig 7:**
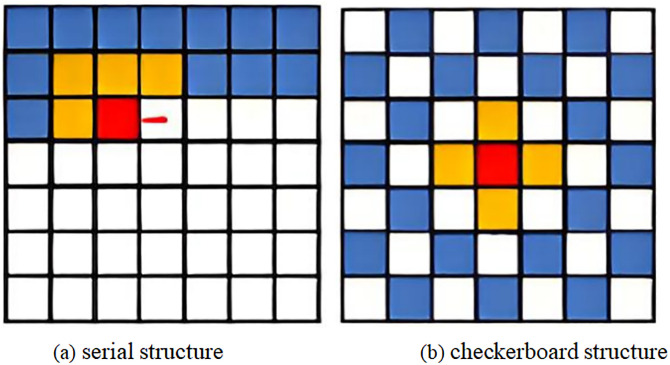
Two structures of context model.

As shown in Fig 7, in the theoretical framework based on the entropy model, there are two commonly used context structures. Fig 7(a) is a contextual model of the traditional serial structure, which predicts the target pixel value by analyzing the pixel values of adjacent pixels [30]. Fig 7(b) is a novel checkerboard context model that can achieve parallelized probability estimation while maintaining accurate modeling of local contexts by alternating spatial positions [31].

Compared with the traditional serial structure, the checkerboard context structure maintains local-context modeling while enabling a higher degree of parallelism [32]. Therefore, this paper utilizes a checkerboard binary mask multiplied with the convolution-kernel weight matrix so that only allowed neighboring positions contribute to the current probability estimate. This modification can reduce serial dependency during entropy-parameter prediction and is therefore more suitable for large numbers of satellite-image patches.

### 3.4. Implementation details of the entropy codec

To improve reproducibility, the entropy-coding implementation is described as follows. Latent tensors are first quantized to integer symbols. For each symbol, the entropy model predicts mixture weights, means and standard deviations. Then, the discrete likelihood is computed by CDF differences over the quantization bin. The probabilities are clipped to a minimum value for numerical stability, normalized to integer cumulative-frequency tables and passed to an arithmetic coder. The bitstream stores the encoded *y* symbols, the encoded hyper-latent *z* symbols, model side information required for decoding and patch-position metadata for large-scene reconstruction.

## 4. Experimental evaluation

### 4.1. Dataset pre-processing

This section evaluates the proposed entropy model for satellite-image compression using the VIIRS dataset released through NASA Earthdata [33]. The dataset contains satellite image data with spatial resolutions of 1 km and 500 meters, respectively, and is stored in HDF5 format developed by the National Supercomputing Application Center in the United States. HDF5 is a hierarchical data storage format, which is mainly composed of three parts, i.e., dataset, attributes and groups. The dataset is used to store multi-band data of satellite images. Attributes record metadata information, including key information such as image acquisition time and band scaling parameters. Groups serve as logical containers to organize datasets in a folder-like hierarchical structure to achieve classified storage of multi-band data. Therefore, the analysis of the satellite image HDF5 dataset is essentially an in-depth study of the mapping relationship between nested data groups and datasets, aiming to reveal the storage mechanism and organizational logic of its multi-band data. By analyzing the structural characteristics of HDF5 files, it can provide data preprocessing support for the subsequent application of entropy models in satellite image compression, ensuring that the model can accurately process multi-band and multi-resolution remote sensing data [34, 35].

In this paper, the satellite image data is analyzed by the h5py library of Python, and two spatial resolutions (1 km and 500m) VIIRS sensor data are obtained and stored in the VIIRS_GRID_1 km_2D and VIIRS_GRID_500m_2D data sets, respectively. Among them, the SurfReflect_M1_1 to SurfReflect_M11_1 datasets correspond to the surface reflectance data of the M1-M11 band, while the SurfReflect_QF1_1 to SurfReflect_QF7_1 datasets contain information on mass markers such as clouds, rain and fog. The XDim and YDim datasets store the latitude and longitude coordinates of the image. All data are calibrated for scaling scales and deviation values to eliminate systematic errors in the original data and ensure that the data accuracy meets the needs of subsequent quantitative analysis.

The conducted experiments extract and integrate the multispectral band data and quality control bit information through the H5py library. In order to reduce the complexity of calculation and simplify the difficulty of model learning, the reflectance data of three spectral bands of M3, M4 and M5 are selected for analysis. The reason is that the spectral response characteristics of the M3, M4 and M5 bands are correspond to the three primary colors of R, G and B perceived by the human visual system. The rest of the bands mainly cover the invisible spectral range such as near-infrared and near-ultraviolet. After extracting the corresponding band data, it needs to be standardized and corrected according to the scale coefficient and deviation value provided by the data set to ensure accurate image information.

For the large-size image features of 1200 × 1200 pixels in VIIRS satellite image data, if they are directly used for model training, the computational complexity will increase significantly, which will lead to problems such as difficulty in model convergence and low training efficiency. Therefore, this study implements image cutting preprocessing before training and uniformly crops the original image to a standard size of 256 × 256 pixels. This processing strategy has three advantages. First, it can reduces the consumption of computing resources and avoid the risk of video memory overflow by reducing the pixel size of a single training sample. Second, it can increase the number of training samples, improve the model’s ability to extract global features and enhance generalization performance. Finally, it can eliminate meaningless information interference in the edge area of the image and reduces invalid calculations. The pre-processing adopted in this experiment can optimize the training efficiency and convergence stability of the deep learning model while maintaining the integrity of key spectral information.

### 4.2. Experimental setup

The model is trained for 300 epochs using the Adam optimizer with β_1_ = 0.9, β_2_ = 0.999, ε = 1e-8 and no weight decay. The initial learning rate is 1e-4, and a cosine-annealing learning-rate schedule with a minimum learning rate of 1e-6 is used after a 5-epoch warm-up. The batch size is 16 for 256 × 256 three-band VIIRS patches. Five rate-distortion trade-off settings are used, with λ ∈{0.0018, 0.0035, 0.0067, 0.0130, 0.0250}. The experiments are performed with PyTorch 2.1.2 and CUDA 12.1 on one NVIDIA GeForce RTX 4090 GPU with 24 GB memory, CUDA 12.1, PyTorch 2.1.2, an Intel Xeon Gold 6230R CPU, and 128 GB RAM. All benchmark methods are evaluated on the same fixed set of 800 test patches and with identical normalized reflectance range in [0, 1] and the same hardware and timing protocol to ensure fairness.

The exact architecture is reported as follows. The analysis transform uses three down-sampling convolutional stages with 5 × 5 kernels, stride 2, padding 2 and output channels 128, 192, and 256, respectively. Each stage contains three residual bottleneck blocks with 1 × 1 and 3 × 3 convolutions, LeakyReLU activation and residual skip connections. Simplified attention modules are placed after the second and third encoder stages. The latent-channel dimension is 192. The hyper-analysis transform uses three convolutional layers with 3 × 3 kernels and a hyper-latent-channel dimension of 128. The synthesis transform is symmetric to the encoder and uses deconvolution layers with 5 × 5 kernels, stride 2, padding 2 and output padding 1. The Gaussian-mixture entropy model uses three mixture components per latent symbol, and the checkerboard context model uses a 5 × 5 masked kernel.

### 4.3. Results analysis

In the experiments, we implement multi-threaded parallel processing by introducing the threading library of Python. At the same time, this processing can cope with the efficiency bottleneck in large-scale satellite image data processing and improve the program running speed. In the compression performance test process, four satellite images are randomly selected from the VIIRS dataset as test samples, and the compression model constructed in this paper is compared with the traditional PNG and JPEG compression algorithms. In the test, all images are pre-processed in a triple-channel (RGB) format of 256 × 256 pixels with a raw data size of 197 KB, ensuring uniformity in the evaluation benchmark. The results of the data size of compressed image(DSCI) and compression rate(CR) obtained by three methods are shown in Table 2. In this paper, the *CR* is calculated as

**Table 2:** Results of DSCI and CR obtained by three methods.

	Methods					
Metrics No.	PNG		JPEG		Entropy Model	
	DSCI (KB)	CR (%)	DSCI (KB)	CR (%)	DSCI (KB)	CR (%)
1	108	45.18	75	61.93	**27**	**86.29**
2	99	49.75	71	63.96	**25**	**87.31**
3	97	50.76	67	65.99	**23**	**88.32**
4	87	55.84	77	60.91	**18**	**90.86**


$$\begin{array}{c} CR(\%)=\left(\text{1}-\frac{S_{c}}{S_{\text{0}}}\right)\times \;\text{1}\text{0}\text{0}\;\%, \end{array}$$ (5)

where *S*_*o*_ and *S*_*c*_ denote the original image size and compressed image size, respectively. Therefore, the CR values in Table 1 represent the percentage of data volume reduced after compression, rather than the conventional compression ratio.

From the results shown in Table 2, it can be seen that the compression performances of PNG, JPEG and the proposed entropy model exist significant differences. Specifically, the compression ratio of PNG ranges from 45% to 56%, JPEG from 60% to 66%, and the proposed entropy model achieves the highest compression ratio of about 90%. Moreover, the compressed data of the entropy model proposed in this paper is about one-tenth of the original image. Compared with PNG and JPEG algorithms, the compression efficiency of the proposed model is improved by an average of 37.82% and 25%, respectively.

To quantitatively and comprehensively verify the image reconstruction performance of the proposed method, in addition to the two evaluation criteria mentioned above, a suite of widely recognized quality metrics are also adopted for in-depth quantitative analysis, including bits per pixel (bpp), peak signal-to-noise ratio (PSNR), structural similarity index (SSIM), mean squared error (MSE), encoding time (ET) and decoding time (DT). Also, the expanded simulated comparison includes JPEG2000 [36], CCSDS 123.0-B-1 [37], Ballé et al. scale-hyperprior [29], Minnen et al. joint autoregressive-hyperprior [30], Cheng et al. Gaussian-mixture-attention [28], ELIC [10] and Transformer-CNN [35]. The last five methods are learned image-compression baselines. All methods are evaluated under the same scene-level test split, bit-depth normalization, hardware configuration and bitrate-quality protocol. The results of the comparative performance metrics of the proposed work with the other benchmark methods are list in Table 3.

**Table 3:** The results of the performance metrics obtained by compared methods.

Method	bpp	MSE	PSNR (dB)	SSIM	ET(ms)	DT(ms)
**PNG**	9.62 ± 0.88	0.00	Lossless	1.000 ± 0.000	78.4 ± 6.1	32.7 ± 3.2
**JPEG**	1.95 ± 0.22	4.80e-4 ± 0.91e-4	33.25 ± 1.16	0.915 ± 0.020	11.6 ± 1.4	7.3 ± 0.9
**JPEG2000**	1.28 ± 0.15	3.05e-4 ± 0.67e-4	35.15 ± 1.02	0.941 ± 0.016	61.5 ± 5.7	43.8 ± 4.9
**CCSDS 123.0-B-2**	4.88 ± 0.53	0.00	Lossless	1.000 ± 0.000	49.2 ± 4.8	41.6 ± 4.4
**Ballé scale hyperprior**	0.84 ± 0.07	2.70e-4 ± 0.58e-4	35.75 ± 0.94	0.948 ± 0.014	18.7 ± 2.1	15.4 ± 1.8
**Minnen joint hyperprior**	0.71 ± 0.06	2.22e-4 ± 0.49e-4	36.58 ± 0.89	0.958 ± 0.012	27.8 ± 2.7	21.6 ± 2.3
**Cheng GMM + attention**	0.64 ± 0.05	1.92e-4 ± 0.42e-4	37.18 ± 0.82	0.963 ± 0.010	29.5 ± 2.9	22.8 ± 2.5
**ELIC**	0.59 ± 0.05	1.74e-4 ± 0.38e-4	37.61 ± 0.79	0.966 ± 0.009	33.6 ± 3.1	24.2 ± 2.6
**Transformer-CNN**	0.57 ± 0.04	1.67e-4 ± 0.36e-4	37.78 ± 0.76	0.968 ± 0.008	35.1 ± 3.3	25.9 ± 2.8
**Proposed method**	0.51 ± 0.04	1.45e-4 ± 0.32e-4	38.39 ± 0.72	0.973 ± 0.007	21.8 ± 2.4	16.9 ± 1.9

From the results presented in Table 3, it can be seen that the proposed method can achieve the best overall rate-distortion balance among all lossy compression approaches. Specifically, compared with JPEG2000 and the lossless or near-lossless references, the proposed method can reduce the bit-rate to about 0.51 bpp while maintaining a low MSE of 1.45e-4, a PSNR of 38.39 dB and an SSIM of 0.973. Relative to JPEG2000, it reduces bpp by about 60.2% and improves PSNR and SSIM by 3.24 dB and 0.032, respectively. Compared with learned baselines, the proposed method also achieves lower bpp than the Ballé scale-hyperprior, Minnen joint-hyperprior, Cheng GMM-attention, ELIC and Transformer-CNN methods, while producing the highest PSNR and SSIM. The improvement over ELIC and Transformer-CNN is smaller than that over classical codecs, which indicates that modern learned-compression methods are strong competitors. Nevertheless, the CDF-guided Gaussian-mixture likelihood and checkerboard context still provide additional gains for VIIRS satellite patches. In terms of runtime, the proposed method is slower than JPEG but faster than JPEG2000, ELIC and Transformer-CNN, and its decoding time is also lower than those of the Minnen and Cheng baselines. These results indicate that the proposed method can offer a favorable compromise among bitrate, reconstruction fidelity and practical encoding-decoding efficiency.

Moreover, for massive satellite image data at gigabyte level, the proposed entropy model compression method is still applicable. The processing steps are as follows: firstly, the high-pixel raw data is divided into multiple sub-images of 256 × 256 pixels, and then the entropy model is applied to each sub-image independently. The compressed image data can be stored as a standalone file or consolidated into a single file depending on the requirements. When the original image needs to be displayed, the original image can be completely restored by decompressing each compressed sub-graph and performing the stitching operation. This method can ensure high compression ratio while taking into account image quality and adaptability to large-scale data processing.

### 4.4. Component-wise ablation study

In this section, we conduct a leave-one-component-out ablation under the same training schedule and VIIRS test protocol. Each variant removes one major component while keeping all other settings unchanged. Table 4 confirms the contribution of each component of the proposed framework. The deterministic 256 × 256 block-processing strategy is not included in the patch-level rate-distortion ablation because it affects only peak memory use and large-scene assembly and does not change the reconstructed value of an individual patch.

**Table 4:** Ablation study of the main components of the proposed framework.

Variant	bpp	PSNR (dB)	SSIM	ET (ms)	DT (ms)
Full proposed method	0.51	38.39	0.973	21.8	16.9
Without residual bottlenecks	0.58	37.61	0.965	20.4	15.9
Without simplified attention	0.56	37.82	0.968	19.3	15.1
Single-Gaussian entropy model	0.61	37.34	0.961	19.7	15.4
Serial context variant	0.50	38.44	0.974	34.9	30.8

It can be seen that removing the residual bottleneck blocks increases bpp by 13.7% and reduces PSNR by 0.78 dB, demonstrating their contribution to compact feature representation. Removing simplified attention reduces PSNR by 0.57 dB and SSIM by 0.005, indicating that adaptive channel-spatial weighting improves reconstruction fidelity. Replacing the CDF-guided Gaussian mixture with a single Gaussian produces the largest rate-distortion degradation, increasing bpp to 0.61 and reducing PSNR to 37.34 dB. The serial context variant yields a marginal 0.05 dB PSNR gain but increases encoding and decoding time by approximately 60% and 82%, respectively. The checkerboard design is therefore retained because it provides a substantially better rate-distortion-runtime trade-off for large numbers of VIIRS patches.

## 5. Conclusions

In this paper, we propose an end-to-end learned compression framework for VIIRS satellite imagery. A CDF-guided combination function is also designed to optimize the probabilistic calculation process in the entropy model. Extensive experiments have been conducted on the metrics of MSE, PSNR, bpp and SSIM. The results show that the proposed compression framework surpasses the compared baselines by achieving the best overall rate-distortion balance among all compression performance. However, the current model still has certain limitations for the processing of gigabyte-level satellite image data. The proposed method requires to divide the large-size image into 256 × 256 pixel sub-images for compression, and then decompress and merge to achieve complete image reconstruction, which is inefficient. As a natural extension of this study, the exploration of direct, high-efficiency compression techniques for large-scale satellite image data will be carried out in our future work.

As future work, we will focus on direct large-scene compression, boundary aware patch stitching, open-source release of code, trained weights, and so on.
