# Supplementary material for: An end-to-end deep learning image compression method for satellite images based on entropy model
Source: PLoS One. 2026 Aug 3;21(8):e0355234. doi: 10.1371/journal.pone.0355234 (PMC13432138; doi:10.1371/journal.pone.0355234)
Supplement: Attachment — Submitted filename: Response to Reviewers.doc [file pone.0355234.s004.doc]

Dear Editor Hashmat Fida,

First of all, we would like to thank you very much for your time and effort in processing our paper entitled *An end-to-end Deep Learning Image Compression Method based on Entropy Model* (PONE-D-26-16540). We would also like to thank the reviewers for their valuable comments and constructive suggestions that help us improve the paper significantly.

As suggested by you, we have thoroughly checked our revised manuscript and made all the necessary changes. Thus we believe the quality and readability of the revised manuscript would be better.

As your requests, we first make the following statements.**1. Role of Funder statement**The funder provided support in the form of salaries for authors [Yanlong Gao, Haiming Xu, Wei Huang and Hao Bai], but did not have any additional role in the study design, data collection and analysis, decision to publish, or preparation of the manuscript. **2. Funding Statement declaring** This does not alter our adherence to PLOS ONE policies on sharing data and materials. Sincerely,

Heyang Xu

We have revised our paper to thoroughly incorporate the reviewers’ comments. In the following we respond to the comments of the reviewers one by one:

**Reviewer #1**

*The authors propose a framework for compressing large-scale remote sensing images using deep learning with an entropy model. The methodology is novel. Paper is well written. Logical flow is consistent throughout the section on the proposed work. However*

*1. They have reported the proposed work only in terms of algorithmic performance and efficiency using metrics such as CR. Suggest to also report distortion and reconstructed image-quality metrics such as PSNR, MSE and Structural Similarity Index Measure (SSIM).*

**Reply 1.1:** Thank you for your comments. Based on your suggestions, we have conducted more experimental studies on the metrics of MSE, PSNR, bpp and SSIM and the results are listed in Table 3. The details can be found in Table 3 and the paragraphs before and after Table 3 in Section 4.3.

*2. Need more baseline comparisons. Performance comparison as of now is done based on the classical transformation techniques such as JPEG and PNG. Suggest anyone of the following:*

*2.1 compare with the official space-mission standards CCSDS 123.0-B-2: which is a benchmark for lossless and near-lossless 3D compression of multispectral and hyperspectral imagery.*

*2.2 State-of-the-art deep learning based diffusion models, on the same data set such as Cosmic or Elic.*

**Reply 1.2:** Thank you for your comments. Based on your suggestions, we have made the following revisions.

**First,** we added more compared baselines including JPEG2000, CCSDS 123.0-B-1, scale-hyperprior, joint autoregressive-hyperprior, Gaussian-mixture-attention, ELIC and Transformer-CNN learned compression models. The details can be found in Table 3 and the paragraphs before and after Table 3 in Section 4.3.

**Second,** we added corresponding references of CCSDS/JPEG2000, i.e., Ref.[36, 37].

**Reviewer #2**

*1.Insufficient Novelty Clarification*

*The proposed framework appears to heavily rely on existing learned image compression architectures such as hyperprior models, attention-based compression, and Gaussian mixture entropy models already introduced in prior works such as Ballé et al., Minnen et al., and Cheng et al. The manuscript does not clearly explain what is fundamentally novel beyond combining known components. The claimed “CDF-based entropy model” is mathematically very similar to standard Gaussian likelihood modeling already widely used in learned image compression literature.*

**Reply 2.1:** Thank you for your comments. Based on your comments, we have made the following revisions.

**First,** we have explicitly explained that scale hyperpriors, attention modules, context models and Gaussian-mixture likelihoods are known components. We clarify that the contribution is their VIIRS-oriented integration, i.e., CDF-based discrete likelihood calculation over quantization bins, checkerboard mask-kernel context implementation, HDF5 multispectral preprocessing, patch-based large-scene compression and an expanded satellite-specific evaluation protocol. The detailed revisions can be found in “Abstract, revised contribution paragraphs in Section 1 and Table 1 Novelty comparison.”

**Second,** in the revised manuscript, the CDF operation is no longer presented as a new probability family. Instead, we explained that CDF differences are used to compute discrete symbol likelihoods for quantized latent variables under a Gaussian-mixture entropy model. We also revised the entropy-model subsection to distinguish adopted prior components from the specific satellite-oriented implementation and evaluation contribution of this manuscript. The detailed revisions can be found in the paragraph before Equation (3) of Section 3.1.

*2. The authors must clearly distinguish their contribution from prior state-of-the-art methods and provide a detailed novelty comparison table.*

**Reply 2.2:** Thank you for your comments. Based on your comments, we have added a paragraph and a comparison table to clearly distinguish our contribution from prior methods. The detailed revisions can be found in Table 1 and the paragraph before Table 1, at the last of Section 1.

*3.Weak Experimental Evaluation*

*The experimental section is insufficient for a high-quality scientific publication.*

*Only four images are used for evaluation.*

*Comparisons are limited to PNG and JPEG, which are outdated baselines for scientific image compression research.*

*No comparison is made with modern learned image compression methods such as:*

*Ballé et al.*

*Cheng et al.*

*ELIC*

*Transformer-based compression methods*

*No rate-distortion curves are presented.*

*No PSNR, SSIM, MS-SSIM, LPIPS, or bitrate evaluation is provided.*

*No ablation study is conducted.*

*Current evidence is not sufficient to support the strong claims regarding superiority and efficiency.*

**Reply 2.3:** Thank you for your comments. Based on your comments, we identified the original four-image result as preliminary and added a full evaluation protocol. Also, the detailed revision can be found in ***Reply 1.2***.

*4. Lack of Statistical Rigor*

*The manuscript does not provide:*

*Statistical significance analysis*

*Multiple-run experiments*

*Variance or standard deviation*

*Confidence intervals*

*Cross-validation strategy*

*The results appear to be based on very limited experiments and therefore cannot be considered statistically rigorous.*

**Reply 2.4:** Thank you for your comments. Based on your comments, we have added a statistical testing protocol requiring mean +/- standard deviation, bootstrap 95% confidence intervals, multiple random seeds and paired significance test for multiple comparisons. The detailed revisions can be found in Table 3 and the paragraphs before and after Table 3 in Section 4.3.

*5. Reproducibility is insufficient*

*The study uses processed NASA VIIRS datasets and generates experimental outputs, but reproducibility is currently insufficient..*

**Reply 2.5:** Thank you for your comments. Based on your comments, we have added a subsection (i.e., Section 4.2 Experimental Setup) to enhance the reproducibility . In this section, we report exact VIIRS granule identifiers, preprocessing scripts, train/validation/test split files, random seeds, trained weights, evaluation scripts, entropy-coding details and a permanent code/data repository link.

*6.Methodological Details Are Missing*

*Important implementation details are absent, including:*

*Number of training epochs*

*Learning rate scheduling*

*Batch size*

*GPU specifications*

*Hyperparameter settings*

*Entropy coding implementation details*

*Exact architecture configurations*

*Computational complexity analysis*

*Inference time benchmarking*

*Without these details, the work is difficult to reproduce.*

**Reply 2.6:** Thank you for your comments. Based on your comments, we have added a reproducibility subsection specifying the items to report, including training epochs, optimizer, learning rate, schedule, batch size, lambda values, seeds, GPU/CUDA/PyTorch versions, architecture parameters, entropy-coding implementation and complexity/runtime measurements. The detailed revision can be found in ***Reply 2.5***.

*7. Questionable Claims*

*Several claims appear exaggerated or insufficiently supported. For example:*

*“10 times faster than PNG and JPEG”*

*“Compression ratio around 90%”*

*“Improved by 37.82% and 25%”*

*These claims require:*

*Proper benchmarking protocols*

*Runtime hardware specifications*

*Statistical validation*

*Fair bitrate-quality comparisons*

**Reply 2.7:** Thank you for your comments. Based on your comments, we have removed the unsupported “10 times faster” claim from the abstract and tones down broad superiority statements in the revised manuscript. Also, the original compression-ratio table is now described as a preliminary smoke test only. The detailed revisions can be found in “Abstract, Section 4.5 Results Analysis, Section 4.7 Computational Complexity and Deployment Analysis, and Conclusion.”

*8. Organization and Consistency Problems*

*There are multiple inconsistencies and possible template-copying errors. For example:*

*Section 1 mentions “VM scheduling” and “FTaVS problem,” which are unrelated to image compression.*

*Several references appear weakly connected to the topic.*

*Some citations seem inappropriate or unrelated to entropy coding.*

*This raises concerns regarding manuscript preparation quality.*

**Reply 2.8:** Thank you for your comments. Based on your comments, we have made the following revisions.

First, we corrected the section title “Instructions” to “Introduction.”

Second, we removed the unrelated “VM scheduling/FTaVS” sentence.

Third, we revised the organization paragraph to match the image-compression topic. Forth, we also corrected obvious reference typo “EEE” to “IEEE” and added relevant baseline references for CCSDS and JPEG2000.

*9. Minor Comments*

*The manuscript contains grammatical issues, excessive general background, insufficient figure analysis, inconsistent notation, a weak conclusion, and should include complexity, memory, GPU utilization, and deployment discussion.*

**Reply 2.9:** Thank you for your comments. Based on your comments, we have made the following revisions.

**First,** we revised the language of the abstract, contribution statements, entropy-model explanation, dataset/evaluation sections, result analysis, deployment discussion and conclusion.

**Second,** we added notation clarification around the CDF likelihood and expanded figure-analysis language. Furthermore, we added complexity, memory and runtime analysis and strengthened the conclusion by explicitly stating limitations and future work.

Some other minor revisions can be found in ***Reply 2.8***.

**Reviewer #3**

*The paper proposes an end-to-end image compression framework based on deep learning entropy model. While the techniques presented are relatively simple, the strength of the paper is in the results, particularly the achieved compression ratio of 10:1 (or 90%) as stated in the results section. Overall the paper is well written, however it requires correcting a few spelling and writing errors.*

*1)- Line 12: "bringing" --> "brings"*

*2)- Line 59: I went over reference [3] and it doesn’t mention any climate models or meteorological applications*

*3)- Line 176: Input image is denoted as “x”, but Fig. 1 shows input image as “X”*

*4)- Line 183: Possible bad formatting: “c” - It does not appear in Fig. 1 either; “y” “y” “z” “z”*

*5)- Fig. 1:*

*- shows g_s outputs y’, but in text you say g_s outputs x’*

*- h_a, h_s are not mentioned in text*

*- it’s not clear what’s the contribution of h_a, h_s to y’*

*6)- Lines 317-318: “a Attention” “an Attention”*

*7)- Line 347: “y” “y”*

*8)- Line 366: “has become” “becomes”*

*9)- Section 2.3: at what stage does uniform scalar quantization occur?*

*10)- Fig. 6: are ga, gs, ha, hs the same as g_a, g_s, h_a, h_s? If so, please use the same notation for consistency*

*11)- Line 420: “is adopt” “is adopted”*

*12)- Line 433: “Si” “Si”*

*13)- Line 526: you call it “compression ratio”, but what you show in Table 1 is actually (1 – 1 / CR) * 100%.*

**Reply 3.1:** Thank you for your comments. Based on your comments, Based on your comments, we have made the following revisions.

1): In original Line 12, "bringing" is revised to "brings".

2): We have replaced reference [3] with a new related work.

3)-5): We have further refined Fig. 1 by explicitly labeling y, y', , z and z' in the corresponding positions of the compression framework. We have also clarified that the quantizer corresponds to the "Q" part of the "Q&CoDec" modules in Fig. 1. In addition, we added a detailed explanation of h_s and h_a. Specifically, h_a is the hyper-analysis transform that extracts the hyper-latent variable z from y, while h_s is the hyper-synthesis transform that estimates the probability distribution parameters of y' based on z'. These two modules constitute the auxiliary hyper-prior branch for entropy modeling rather than the main encoder and decoder. The detailed revisions can be found in the first paragraph after Fig. 1.

6): In original Lines 317-318, “a Attention” is revised to “an Attention”.

7): In original Line 347, “y” is revised as “*y*”.

8): In original Line 366, “has become” is revised to “becomes”.

9): In Section 2.3, the proposed uniform scalar quantization is used in the latent quantization stage, specifically in both the main latent representation *y* generated by g_a and the hyper-latent representation *z* generated by h_a. We have added the explanation in the paragraph before Equation (2).

10) : Thank you for pointing this out. The symbols "ga, gs, ha, hs" in fig. 6 have the same meanings as g_a, g_s, h_a and h_s. To avoid ambiguity, we have revised Fig. 6 and unified all symbols as g_a, g_s, h_a and h_s.

11): In original Line 420, “is adopt” is revised as “is adopted”.

12): In original Line 433, “Si” is revised as “*S_i_*”.

13): Thank you for this careful comment. We agree that the CR values in Table 1 do not correspond to the conventional compression ratio *S_o_*/*S_c_*. Instead, they represent the percentage of data volume reduced after compression, calculated as *CR*(%)=(1-*S_c_*/*S_o_*)* 100%. Therefore, we have revised the text before Table 1 by replacing "compression ratio" with "compression rate". We have also clarified the calculation formula and modified the table header accordingly to avoid confusion. The detailed revisions can be found in the paragraph before Table 1.

And some other changes are made which are not listed here for the sake of brevity.
