# Supplementary material for: An end-to-end deep learning image compression method for satellite images based on entropy model
Source: PLoS One. 2026 Aug 3;21(8):e0355234. doi: 10.1371/journal.pone.0355234 (PMC13432138; doi:10.1371/journal.pone.0355234)
Supplement: Attachment — Submitted filename: Response_to_Reviewers_auresp_2.doc [file pone.0355234.s005.doc]

Dear Editor Hashmat Fida,

First of all, we would like to thank you very much for your time and effort in processing our paper entitled *An end-to-end Deep Learning Image Compression Method based on Entropy Model* (PONE-D-26-16540). We would also like to thank the reviewers for their valuable comments and constructive suggestions that help us improve the paper significantly.

As suggested by you, we have thoroughly checked our revised manuscript and made all the necessary changes. Thus we believe the quality and readability of the revised manuscript would be better.

Sincerely,

Heyang Xu

We once again thank the reviewers for their valuable comments. We have revised our paper to thoroughly incorporate the reviewers’ comments. In the following we respond to the comments of the reviewers one by one:

**Reviewer #1**

*1. Confirm that the same randomly selected images from the VIIRS dataset were used across all compared methods to ensure a fair comparison.*

**Reply 1.1:** Thank you for your comments. Based on your suggestions, we have conducted more experimental studies on the metrics of MSE, PSNR, bpp and SSIM and the results are listed in Table 3. The details can be found in Table 3 and the paragraphs before and after Table 3 in Section 4.3.

*2. Include a brief summary of the comparative performance with existing methods in the Abstract.*

**Reply 1.2:** Thank you for your comments. Based on your suggestions, we have added a brief summary of the comparative performance with existing methods in the Abstract. The added summary is “....the proposed method achieves 0.51±0.04 bpp, 38.39±0.72 dB PSNR and 0.973±0.007 SSIM. Relative to ELIC and the Transformer-CNN baseline, it reduced bpp by 13.6% and 10.5% and improved PSNR by 0.78 dB and 0.61 dB, respectively.”

*3.Revise the caption and the corresponding in-text reference for Table 3 to clarify that it summarizes comparisons with benchmark methods rather than benchmark datasets.*

**Reply 1.3:** Thank you for your comments. Based on your comments, we have revised the caption of Table 3 and the corresponding in-text reference. The detailed revisions are as follows:

**First,** the caption of Table 3 is revised to “Table 3. The results of the performance metrics obtained by compared methods.”

**Second,** the corresponding in-text reference for Table 3 is revised to “The results of the comparative performance metrics of the proposed work with the other benchmark methods are list in Table 3.”

*4. Correct the language issues identified in Lines 598, 618, and 732, along with any remaining grammatical or typographical errors throughout the manuscript.*

**Reply 1.4:** Thank you for your comments. Based on your suggestions, we have carefully proofread and made the following revisions.

1): The sentence “The processing process is as follows----” is revised to “The processing steps are as follows-----.”

2): The last sentence in the first paragraph of Section “5. Conclusions” is revised to “As a natural extension of this study, the exploration of direct, high-efficiency compression techniques for large-scale satellite image data will be carried out in our future work.”

3): The word “vailable” in Reference [37] is revised to “available”.

4): The word “Satelllite” in the title of the paper is revised to “Satellite”.

And some other changes are made which are not listed here for the sake of brevity.

**Reviewer #2**

*1.Add a dedicated novelty comparison table that clearly distinguishes the proposed method from recent learned image compression approaches, including Ballé, Minnen, Cheng, ELIC, and Transformer-based methods.*

**Reply 2.1:** Thank you for your comments. Based on your comments, we have reorganized Table 1.

*2. Include comparisons with relevant state-of-the-art learned image compression methods or provide a clear justification if such comparisons cannot be performed.*

**Reply 2.2:** Thank you for your comments. Based on your comments, the comparative evaluation has been broadened to include representative modern learned-compression baselines, including Ballé scale hyperprior, Minnen joint autoregressive-hyperprior, Cheng Gaussian-mixture-attention, ELIC and a mixed Transformer-CNN method. To avoid confusion, we have explicitly pointed this out in the paragraph before Table 3. Also, in the discussion paragraph after Table 3, we acknowledge that the margins over recent learned methods are smaller than those over classical codecs.

*3.Provide a component-wise ablation study demonstrating the contribution of each major module in the proposed framework. If an ablation study is not feasible, please explain the reason in the manuscript.*

**Reply 2.3:** Thank you for your comments. Based on your comments, we have added a component-wise leave-one-out ablation section to isolate the effects of the residual bottleneck blocks, simplified attention modules, Gaussian-mixture entropy model and checkerboard context. The detailed revisions can be in Section “4. 4 Component-Wise Ablation Study.”

*4. Explicitly report the batch size and any other important training parameters used during model development to improve reproducibility.*

**Reply 2.4:** Thank you for your comments. Based on your comments, we have expanded the experimental setup to include the batch size, optimizer parameters, learning-rate schedule, number of epochs, rate-distortion weights, software versions, hardware, test-set size and so on. The detailed revisions can be found in Section “4.2 Experimental Setup.”

We again thank the reviewers for their constructive comments, which have substantially improved the clarity and technical completeness of the manuscript.
