# Supplementary material for: An end-to-end deep learning image compression method for satellite images based on entropy model
Source: PLoS One. 2026 Aug 3;21(8):e0355234. doi: 10.1371/journal.pone.0355234 (PMC13432138; doi:10.1371/journal.pone.0355234)
Supplement: Attachment — Submitted filename: PONE-D-26-16540-review.docx [file pone.0355234.s001.docx]

**Some comments:**

- Line 12: "bringing" --> "brings"

- Line 59: I went over reference [3] and it doesn’t mention any climate models or meteorological applications

- Line 176: Input image is denoted as “*x*”, but Fig. 1 shows input image as “X”

- Line 183: Possible bad formatting: “c” - It does not appear in Fig. 1 either; “y” 🡪 “*y*” “z” 🡪 “*z*”

- Fig. 1:

- shows *g_s* outputs *y’*, but in text you say *g_s* outputs *x’*

- *h_a, h_s* are not mentioned in text

- it’s not clear what’s the contribution of *h_a, h_s* to *y’*

- Lines 317-318: “a Attention” 🡪 “an Attention”

- Line 347: “y” 🡪 “*y*”

- Line 366: “has become” 🡪 “becomes”

- Section 2.3: at what stage does uniform scalar quantization occur?

- Fig. 6: are *g_a_*, *g_s_*, *h_a_*, *h_s_* the same as *g_a, g_s, h_a, h_s*? If so, please use the same notation for consistency

- Line 420: “is adopt” 🡪 “is adopted”

- Line 433: “Si” 🡪 “*S_i_*”

- Line 526: you call it “compression ratio”, but what you show in Table 1 is actually (1 – 1 / CR) * 100%
