# Supplementary material for: An end-to-end deep learning image compression method for satellite images based on entropy model
Source: PLoS One. 2026 Aug 3;21(8):e0355234. doi: 10.1371/journal.pone.0355234 (PMC13432138; doi:10.1371/journal.pone.0355234)
Supplement: Attachment — Submitted filename: PLOS_One_round2_review.docx [file pone.0355234.s003.docx]

Round 2 review: 11.07.26

The authors have sincerely incorporated the suggestions given in my previous round of review. Compared the proposed work with other state of the art methods and included the suggested performance metrics. The new Table 3 states the same.

However, the authors have to confirm that the same set of randomly chosen images from the VIIRS data set is used with all the models for the comparison.

Minor comments:

1. A summary of the comparative performance with existing methods, should also be included in the abstract.
2. Line no. 578, the caption of Table 3 and its in-text reference needs to be changed. The table is a summary of the comparative performance metrics of the proposed work with the other “benchmarking methods” and “not benchmarking data sets”..
3. Line No. 598 --- sentence formation has to be corrected – “The processing process is as follows----”
4. Line No..618 ---sentence formation is very lucid –” Therefore, how to achieve direct and efficient compression….”
5. Line No.732 .. correct the word “vailable…”
