# Supplementary material for: An end-to-end deep learning image compression method for satellite images based on entropy model
Source: PLoS One. 2026 Aug 3;21(8):e0355234. doi: 10.1371/journal.pone.0355234 (PMC13432138; doi:10.1371/journal.pone.0355234)
Supplement: Peer review history

# Peer review: Decision Letter 0

**DOI:** 10.1371/journal.pone.0355234.r001

**Authors:** Hashmat Fida

27 May 2026

PONE-D-26-16540

An end-to-end Deep Learning Image Compression Method based on Entropy Model

PLOS One

Dear Dr. Xu,

Thank you for submitting your manuscript to PLOS ONE. After careful consideration, we feel that it has merit but does not fully meet PLOS ONE’s publication criteria as it currently stands. Therefore, we invite you to submit a revised version of the manuscript that addresses the points raised during the review process.

Please submit your revised manuscript by Jul 11 2026 11:59PM. If you will need more time than this to complete your revisions, please reply to this message or contact the journal office at plosone@plos.org. When you're ready to submit your revision, log on to https://www.editorialmanager.com/pone/ and select the 'Submissions Needing Revision' folder to locate your manuscript file.

Please include the following items when submitting your revised manuscript:

- A letter that responds to each point raised by the academic editor and reviewer(s). You should upload this letter as a separate file labeled 'Response to Reviewers'.
- A marked-up copy of your manuscript that highlights changes made to the original version. You should upload this as a separate file labeled 'Revised Manuscript with Track Changes'.
- An unmarked version of your revised paper without tracked changes. You should upload this as a separate file labeled 'Manuscript'.

If you would like to make changes to your financial disclosure, please include your updated statement in your cover letter. Guidelines for resubmitting your figure files are available below the reviewer comments at the end of this letter.

If applicable, we recommend that you deposit your laboratory protocols in protocols.io to enhance the reproducibility of your results. Protocols.io assigns your protocol its own identifier (DOI) so that it can be cited independently in the future. For instructions see: https://journals.plos.org/plosone/s/submission-guidelines#loc-laboratory-protocols. Additionally, PLOS ONE offers an option for publishing peer-reviewed Lab Protocol articles, which describe protocols hosted on protocols.io. Read more information on sharing protocols at https://plos.org/protocols?utm_medium=editorial-email&utm_source=authorletters&utm_campaign=protocols.

As the corresponding author, your ORCID iD is verified in the submission system and will appear in the published article. PLOS supports the use of ORCID, and we encourage all coauthors to register for an ORCID iD and use it as well. Please encourage your coauthors to verify their ORCID iD within the submission system before final acceptance, as unverified ORCID iDs will not appear in the published article. *Only*  the individual author can complete the verification step; PLOS staff *cannot*  verify ORCID iDs on behalf of authors.

We look forward to receiving your revised manuscript.

Kind regards,

Hashmat Fida

Guest Editor

PLOS One

Journal Requirements:

When submitting your revision, we need you to address these additional requirements.

1.Please ensure that your manuscript meets PLOS ONE's style requirements, including those for file naming. The PLOS ONE style templates can be found at

https://journals.plos.org/plosone/s/file?id=wjVg/PLOSOne_formatting_sample_main_body.pdf and

https://journals.plos.org/plosone/s/file?id=ba62/PLOSOne_formatting_sample_title_authors_affiliations.pdf

2. Please note that PLOS One has specific guidelines on code sharing for submissions in which author-generated code underpins the findings in the manuscript. In these cases, we expect all author-generated code to be made available without restrictions upon publication of the work. Please review our guidelines at https://journals.plos.org/plosone/s/materials-and-software-sharing#loc-sharing-code and ensure that your code is shared in a way that follows best practice and facilitates reproducibility and reuse.

3. Please note that funding information should not appear in any section or other areas of your manuscript. We will only publish funding information present in the Funding Statement section of the online submission form. Please remove any funding-related text from the manuscript.

4. We note that the grant information you provided in the ‘Funding Information’ and ‘Financial Disclosure’ sections do not match.

When you resubmit, please ensure that you provide the correct grant numbers for the awards you received for your study in the ‘Funding Information’ section.

5. Thank you for stating the following financial disclosure:

“the Project of Natural science project of Sichuan Expressway (No. ChuanGao2025-cg-ky-8)”

Please state what role the funders took in the study.  If the funders had no role, please state: "The funders had no role in study design, data collection and analysis, decision to publish, or preparation of the manuscript."

If this statement is not correct you must amend it as needed.

Please include this amended Role of Funder statement in your cover letter; we will change the online submission form on your behalf.

6. Please provide a complete Data Availability Statement in the submission form, ensuring you include all necessary access information or a reason for why you are unable to make your data freely accessible. If your research concerns only data provided within your submission, please write "All data are in the manuscript and/or supporting information files" as your Data Availability Statement.

7. Thank you for stating the following in the Competing Interests section:

“The authors have declared that no competing interests exist.”

We note that one or more of the authors are employed by a commercial company: Sichuan Expressway Construction and Development Group Co., Ltd, Chengdu, Sichuan 610041, China and Sichuan Panyan Expressway Construction and Development Co., Ltd, Chengdu, Sichuan 610041, China

1.        Please provide an amended Funding Statement declaring this commercial affiliation, as well as a statement regarding the Role of Funders in your study. If the funding organization did not play a role in the study design, data collection and analysis, decision to publish, or preparation of the manuscript and only provided financial support in the form of authors' salaries and/or research materials, please review your statements relating to the author contributions, and ensure you have specifically and accurately indicated the role(s) that these authors had in your study. You can update author roles in the Author Contributions section of the online submission form.

Please also include the following statement within your amended Funding Statement.

“The funder provided support in the form of salaries for authors [insert relevant initials], but did not have any additional role in the study design, data collection and analysis, decision to publish, or preparation of the manuscript. The specific roles of these authors are articulated in the ‘author contributions’ section.”

If your commercial affiliation did play a role in your study, please state and explain this role within your updated Funding Statement.

2. Please also provide an updated Competing Interests Statement declaring this commercial affiliation along with any other relevant declarations relating to employment, consultancy, patents, products in development, or marketed products, etc.

Within your Competing Interests Statement, please confirm that this commercial affiliation does not alter your adherence to all PLOS ONE policies on sharing data and materials by including the following statement: "This does not alter our adherence to  PLOS ONE policies on sharing data and materials.” (as detailed online in our guide for authors http://journals.plos.org/plosone/s/competing-interests) . If this adherence statement is not accurate and  there are restrictions on sharing of data and/or materials, please state these. Please note that we cannot proceed with consideration of your article until this information has been declared.

Please include both an updated Funding Statement and Competing Interests Statement in your cover letter. We will change the online submission form on your behalf.

8. We note that Figure 8 in your submission contain copyrighted images. All PLOS content is published under the Creative Commons Attribution License (CC BY 4.0), which means that the manuscript, images, and Supporting Information files will be freely available online, and any third party is permitted to access, download, copy, distribute, and use these materials in any way, even commercially, with proper attribution. For more information, see our copyright guidelines: http://journals.plos.org/plosone/s/licenses-and-copyright.

We require you to either (1) present written permission from the copyright holder to publish these figures specifically under the CC BY 4.0 license, or (2) remove the figures from your submission:

1. You may seek permission from the original copyright holder of Figure 8 to publish the content specifically under the CC BY 4.0 license.

We recommend that you contact the original copyright holder with the Content Permission Form (http://journals.plos.org/plosone/s/file?id=7c09/content-permission-form.pdf) and the following text:

“I request permission for the open-access journal PLOS ONE to publish XXX under the Creative Commons Attribution License (CCAL) CC BY 4.0 (http://creativecommons.org/licenses/by/4.0/). Please be aware that this license allows unrestricted use and distribution, even commercially, by third parties. Please reply and provide explicit written permission to publish XXX under a CC BY license and complete the attached form.”

Please upload the completed Content Permission Form or other proof of granted permissions as an "Other" file with your submission.

In the figure caption of the copyrighted figure, please include the following text: “Reprinted from [ref] under a CC BY license, with permission from [name of publisher], original copyright [original copyright year].”

2. If you are unable to obtain permission from the original copyright holder to publish these figures under the CC BY 4.0 license or if the copyright holder’s requirements are incompatible with the CC BY 4.0 license, please either i) remove the figure or ii) supply a replacement figure that complies with the CC BY 4.0 license. Please check copyright information on all replacement figures and update the figure caption with source information. If applicable, please specify in the figure caption text when a figure is similar but not identical to the original image and is therefore for illustrative purposes only.

9. If the reviewer comments include a recommendation to cite specific previously published works, please review and evaluate these publications to determine whether they are relevant and should be cited. There is no requirement to cite these works unless the editor has indicated otherwise.

Additional Editor Comments:

Dear Authors,

The reviewers have completed the evaluation of your manuscript titled “An end-to-end Deep Learning Image Compression Method based on Entropy Model.” Based on the reviewers’ comments, the manuscript requires major revisions before it can be considered further.

Please revise the manuscript carefully according to the reviewers’ suggestions and submit the revised version along with a detailed response to the comments.

Best regards,

Guest Editor

[Note: HTML markup is below. Please do not edit.]

Reviewers' comments:

Reviewer's Responses to Questions

**Comments to the Author**

1. Is the manuscript technically sound, and do the data support the conclusions?

The manuscript must describe a technically sound piece of scientific research with data that supports the conclusions. Experiments must have been conducted rigorously, with appropriate controls, replication, and sample sizes. The conclusions must be drawn appropriately based on the data presented.

Reviewer #1: Partly

Reviewer #2: Partly

Reviewer #3: Yes

**********

2. Has the statistical analysis been performed appropriately and rigorously?

Reviewer #1: N/A

Reviewer #2: No

Reviewer #3: Yes

**********

3. Have the authors made all data underlying the findings in their manuscript fully available?

The PLOS Data policy requires authors to make all data underlying the findings described in their manuscript fully available without restriction, with rare exception (please refer to the Data Availability Statement in the manuscript PDF file). The data should be provided as part of the manuscript or its supporting information, or deposited to a public repository. For example, in addition to summary statistics, the data points behind means, medians and variance measures should be available. If there are restrictions on publicly sharing data—e.g. participant privacy or use of data from a third party—those must be specified.

Reviewer #1: Yes

Reviewer #2: No

Reviewer #3: Yes

**********

4. Is the manuscript presented in an intelligible fashion and written in standard English?

PLOS ONE does not copyedit accepted manuscripts, so the language in submitted articles must be clear, correct, and unambiguous. Any typographical or grammatical errors should be corrected at revision, so please note any specific errors here.

Reviewer #1: Yes

Reviewer #2: No

Reviewer #3: Yes

**********

5. Review Comments to the Author

Please use the space provided to explain your answers to the questions above. You may also include additional comments for the author, including concerns about dual publication, research ethics, or publication ethics. (Please upload your review as an attachment if it exceeds 20,000 characters)

Reviewer #1: The authors propose a framework for compressing large-scale remote sensing images using deep learning with an entropy model. The methodology is novel. Paper is well written. Logical flow is consistent throughout the section on the proposed work.

However

1. They have reported the proposed work only in terms of algorithmic performance and efficiency using metrics such as CR. Suggest to also report distortion and reconstructed image-quality metrics such as PSNR, MSE and Structural Similarity Index Measure (SSIM).

2. Need more baseline comparisons. Performance comparison as of now is done based on the classical transformation techniques such as JPEG and PNG. Suggest anyone of the following:

2.1 compare with the official space-mission standards CCSDS 123.0-B-2: which is a benchmark for lossless and near-lossless 3D compression of multispectral and hyperspectral imagery.

2.2 State-of-the-art deep learning based diffusion models, on the same data set such as Cosmic or Elic.

Reviewer #2: he manuscript entitled “An end-to-end Deep Learning Image Compression Method based on Entropy Model” proposes a deep learning framework for satellite image compression using an entropy model based on cumulative distribution functions (CDFs). The topic is relevant to remote sensing and large-scale satellite data processing. The manuscript also attempts to integrate encoder–decoder architectures, attention mechanisms, and entropy coding into an end-to-end compression framework.

However, although the paper presents an interesting application direction, the current manuscript has several significant technical, experimental, and presentation-related limitations. Therefore, I recommend major revision before the manuscript can be considered for publication.

Major Comments

Insufficient Novelty Clarification

The proposed framework appears to heavily rely on existing learned image compression architectures such as hyperprior models, attention-based compression, and Gaussian mixture entropy models already introduced in prior works such as Ballé et al., Minnen et al., and Cheng et al. The manuscript does not clearly explain what is fundamentally novel beyond combining known components. The claimed “CDF-based entropy model” is mathematically very similar to standard Gaussian likelihood modeling already widely used in learned image compression literature.

The authors must clearly distinguish their contribution from prior state-of-the-art methods and provide a detailed novelty comparison table.

Weak Experimental Evaluation

Final Recommendation

The paper has potential, but the current version lacks sufficient experimental rigor, novelty clarification, reproducibility, and writing quality required for publication. I therefore recommend Major Revision. Significant improvements in experiments, comparisons, technical clarity, and manuscript presentation are required before reconsideration.

The experimental section is insufficient for a high-quality scientific publication.

Only four images are used for evaluation.

Comparisons are limited to PNG and JPEG, which are outdated baselines for scientific image compression research.

No comparison is made with modern learned image compression methods such as:

Ballé et al.

Cheng et al.

ELIC

Transformer-based compression methods

No rate-distortion curves are presented.

No PSNR, SSIM, MS-SSIM, LPIPS, or bitrate evaluation is provided.

No ablation study is conducted.

Current evidence is not sufficient to support the strong claims regarding superiority and efficiency.

Lack of Statistical Rigor

The manuscript does not provide:

Statistical significance analysis

Multiple-run experiments

Variance or standard deviation

Confidence intervals

Cross-validation strategy

The results appear to be based on very limited experiments and therefore cannot be considered statistically rigorous.

Data Availability Issue

The manuscript states:

“No data was generated by this study.”

However, the study clearly uses processed NASA VIIRS datasets and generates experimental outputs. The authors should provide:

Dataset access links

Preprocessing scripts

Model training configuration

Source code

Compression/decompression implementation details

Reproducibility is currently insufficient.

Methodological Details Are Missing

Important implementation details are absent, including:

Number of training epochs

Learning rate scheduling

Batch size

GPU specifications

Hyperparameter settings

Entropy coding implementation details

Exact architecture configurations

Computational complexity analysis

Inference time benchmarking

Without these details, the work is difficult to reproduce.

Questionable Claims

Several claims appear exaggerated or insufficiently supported. For example:

“10 times faster than PNG and JPEG”

“Compression ratio around 90%”

“Improved by 37.82% and 25%”

These claims require:

Proper benchmarking protocols

Runtime hardware specifications

Statistical validation

Fair bitrate-quality comparisons

Organization and Consistency Problems

There are multiple inconsistencies and possible template-copying errors. For example:

Section 1 mentions “VM scheduling” and “FTaVS problem,” which are unrelated to image compression.

Several references appear weakly connected to the topic.

Some citations seem inappropriate or unrelated to entropy coding.

This raises concerns regarding manuscript preparation quality.

Minor Comments

The manuscript contains many grammatical and language issues. Extensive English editing is necessary.

The paper repeatedly explains general deep learning concepts that are already well known and should instead focus more on the proposed contribution.

Figures are referenced but not sufficiently analyzed.

The notation is inconsistent in several equations.

The conclusion section is relatively weak and mostly repeats earlier statements.

The manuscript should include:

Complexity analysis

Memory consumption

GPU utilization

Real-world deployment discussion

Reviewer #3: The paper proposes an end-to-end image compression framework based on deep learning entropy model. While the techniques presented are relatively simple, the strength of the paper is in the results, particularly the achieved compression ratio of 10:1 (or 90%) as stated in the results section.

Overall the paper is well written, however it requires correcting a few spelling and writing errors (attached).

**********

6. PLOS authors have the option to publish the peer review history of their article (what does this mean?). If published, this will include your full peer review and any attached files.

If you choose “no”, your identity will remain anonymous but your review may still be made public.

**Do you want your identity to be public for this peer review?**  For information about this choice, including consent withdrawal, please see our Privacy Policy.

Reviewer #1: No

Reviewer #2: No

Reviewer #3: No

**********

[NOTE: If reviewer comments were submitted as an attachment file, they will be attached to this email and accessible via the submission site. Please log into your account, locate the manuscript record, and check for the action link "View Attachments". If this link does not appear, there are no attachment files.]

To ensure your figures meet our technical requirements, please review our figure guidelines: https://journals.plos.org/plosone/s/figures

You may also use PLOS’s free figure tool, NAAS, to help you prepare publication quality figures: https://journals.plos.org/plosone/s/figures#loc-tools-for-figure-preparation.

NAAS will assess whether your figures meet our technical requirements by comparing each figure against our figure specifications.

Attachment Submitted filename: PONE-D-26-16540-review.docx

# Author comment: Author response to Decision Letter 1

**DOI:** 10.1371/journal.pone.0355234.r002

8 Jul 2026

We have revised our paper to thoroughly incorporate the reviewers’ comments. In the following we respond to the comments of the reviewers one by one:

Reviewer #1

The authors propose a framework for compressing large-scale remote sensing images using deep learning with an entropy model. The methodology is novel. Paper is well written. Logical flow is consistent throughout the section on the proposed work. However

1. They have reported the proposed work only in terms of algorithmic performance and efficiency using metrics such as CR. Suggest to also report distortion and reconstructed image-quality metrics such as PSNR, MSE and Structural Similarity Index Measure (SSIM).

Reply 1.1: Thank you for your comments. Based on your suggestions, we have conducted more experimental studies on the metrics of MSE, PSNR, bpp and SSIM and the results are listed in Table 3. The details can be found in Table 3 and the paragraphs before and after Table 3 in Section 4.3.

2. Need more baseline comparisons. Performance comparison as of now is done based on the classical transformation techniques such as JPEG and PNG. Suggest anyone of the following:

2.1 compare with the official space-mission standards CCSDS 123.0-B-2: which is a benchmark for lossless and near-lossless 3D compression of multispectral and hyperspectral imagery.

2.2 State-of-the-art deep learning based diffusion models, on the same data set such as Cosmic or Elic.

Reply 1.2: Thank you for your comments. Based on your suggestions, we have made the following revisions.

First, we added more compared baselines including JPEG2000, CCSDS 123.0-B-1, scale-hyperprior, joint autoregressive-hyperprior, Gaussian-mixture-attention, ELIC and Transformer-CNN learned compression models. The details can be found in Table 3 and the paragraphs before and after Table 3 in Section 4.3.

Second, we added corresponding references of CCSDS/JPEG2000, i.e., Ref.[36, 37].

Reviewer #2

1.Insufficient Novelty Clarification

The proposed framework appears to heavily rely on existing learned image compression architectures such as hyperprior models, attention-based compression, and Gaussian mixture entropy models already introduced in prior works such as Ballé et al., Minnen et al., and Cheng et al. The manuscript does not clearly explain what is fundamentally novel beyond combining known components. The claimed “CDF-based entropy model” is mathematically very similar to standard Gaussian likelihood modeling already widely used in learned image compression literature.

Reply 2.1: Thank you for your comments. Based on your comments, we have made the following revisions.

First, we have explicitly explained that scale hyperpriors, attention modules, context models and Gaussian-mixture likelihoods are known components. We clarify that the contribution is their VIIRS-oriented integration, i.e., CDF-based discrete likelihood calculation over quantization bins, checkerboard mask-kernel context implementation, HDF5 multispectral preprocessing, patch-based large-scene compression and an expanded satellite-specific evaluation protocol. The detailed revisions can be found in “Abstract, revised contribution paragraphs in Section 1 and Table 1 Novelty comparison.”

Second, in the revised manuscript, the CDF operation is no longer presented as a new probability family. Instead, we explained that CDF differences are used to compute discrete symbol likelihoods for quantized latent variables under a Gaussian-mixture entropy model. We also revised the entropy-model subsection to distinguish adopted prior components from the specific satellite-oriented implementation and evaluation contribution of this manuscript. The detailed revisions can be found in the paragraph before Equation (3) of Section 3.1.

2. The authors must clearly distinguish their contribution from prior state-of-the-art methods and provide a detailed novelty comparison table.

Reply 2.2: Thank you for your comments. Based on your comments, we have added a paragraph and a comparison table to clearly distinguish our contribution from prior methods. The detailed revisions can be found in Table 1 and the paragraph before Table 1, at the last of Section 1.

3.Weak Experimental Evaluation

The experimental section is insufficient for a high-quality scientific publication.

Only four images are used for evaluation.

Comparisons are limited to PNG and JPEG, which are outdated baselines for scientific image compression research.

No comparison is made with modern learned image compression methods such as:

Ballé et al.

Cheng et al.

ELIC

Transformer-based compression methods

No rate-distortion curves are presented.

No PSNR, SSIM, MS-SSIM, LPIPS, or bitrate evaluation is provided.

No ablation study is conducted.

Current evidence is not sufficient to support the strong claims regarding superiority and efficiency.

Reply 2.3: Thank you for your comments. Based on your comments, we identified the original four-image result as preliminary and added a full evaluation protocol. Also, the detailed revision can be found in Reply 1.2.

4. Lack of Statistical Rigor

The manuscript does not provide:

Statistical significance analysis

Multiple-run experiments

Variance or standard deviation

Confidence intervals

Cross-validation strategy

The results appear to be based on very limited experiments and therefore cannot be considered statistically rigorous.

Reply 2.4: Thank you for your comments. Based on your comments, we have added a statistical testing protocol requiring mean +/- standard deviation, bootstrap 95% confidence intervals, multiple random seeds and paired significance test for multiple comparisons. The detailed revisions can be found in Table 3 and the paragraphs before and after Table 3 in Section 4.3.

5. Reproducibility is insufficient

The study uses processed NASA VIIRS datasets and generates experimental outputs, but reproducibility is currently insufficient..

Reply 2.5: Thank you for your comments. Based on your comments, we have added a subsection (i.e., Section 4.2 Experimental Setup) to enhance the reproducibility . In this section, we report exact VIIRS granule identifiers, preprocessing scripts, train/validation/test split files, random seeds, trained weights, evaluation scripts, entropy-coding details and a permanent code/data repository link.

6.Methodological Details Are Missing

Important implementation details are absent, including:

Number of training epochs

Learning rate scheduling

Batch size

GPU specifications

Hyperparameter settings

Entropy coding implementation details

Exact architecture configurations

Computational complexity analysis

Inference time benchmarking

Without these details, the work is difficult to reproduce.

Reply 2.6: Thank you for your comments. Based on your comments, we have added a reproducibility subsection specifying the items to report, including training epochs, optimizer, learning rate, schedule, batch size, lambda values, seeds, GPU/CUDA/PyTorch versions, architecture parameters, entropy-coding implementation and complexity/runtime measurements. The detailed revision can be found in Reply 2.5.

7. Questionable Claims

Several claims appear exaggerated or insufficiently supported. For example:

“10 times faster than PNG and JPEG”

“Compression ratio around 90%”

“Improved by 37.82% and 25%”

These claims require:

Proper benchmarking protocols

Runtime hardware specifications

Statistical validation

Fair bitrate-quality comparisons

Reply 2.7: Thank you for your comments. Based on your comments, we have removed the unsupported “10 times faster” claim from the abstract and tones down broad superiority statements in the revised manuscript. Also, the original compression-ratio table is now described as a preliminary smoke test only. The detailed revisions can be found in “Abstract, Section 4.5 Results Analysis, Section 4.7 Computational Complexity and Deployment Analysis, and Conclusion.”

8. Organization and Consistency Problems

There are multiple inconsistencies and possible template-copying errors. For example:

Section 1 mentions “VM scheduling” and “FTaVS problem,” which are unrelated to image compression.

Several references appear weakly connected to the topic.

Some citations seem inappropriate or unrelated to entropy coding.

This raises concerns regarding manuscript preparation quality.

Reply 2.8: Thank you for your comments. Based on your comments, we have made the following revisions.

First, we corrected the section title “Instructions” to “Introduction.”

Second, we removed the unrelated “VM scheduling/FTaVS” sentence.

Third, we revised the organization paragraph to match the image-compression topic. Forth, we also corrected obvious reference typo “EEE” to “IEEE” and added relevant baseline references for CCSDS and JPEG2000.

9. Minor Comments

The manuscript contains grammatical issues, excessive general background, insufficient figure analysis, inconsistent notation, a weak conclusion, and should include complexity, memory, GPU utilization, and deployment discussion.

Reply 2.9: Thank you for your comments. Based on your comments, we have made the following revisions.

First, we revised the language of the abstract, contribution statements, entropy-model explanation, dataset/evaluation sections, result analysis, deployment discussion and conclusion.

Second, we added notation clarification around the CDF likelihood and expanded figure-analysis language. Furthermore, we added complexity, memory and runtime analysis and strengthened the conclusion by explicitly stating limitations and future work.

Some other minor revisions can be found in Reply 2.8.

Reviewer #3

The paper proposes an end-to-end image compression framework based on deep learning entropy model. While the techniques presented are relatively simple, the strength of the paper is in the results, particularly the achieved compression ratio of 10:1 (or 90%) as stated in the results section. Overall the paper is well written, however it requires correcting a few spelling and writing errors.

1)- Line 12: "bringing" --> "brings"

2)- Line 59: I went over reference [3] and it doesn’t mention any climate models or meteorological applications

3)- Line 176: Input image is denoted as “x”, but Fig. 1 shows input image as “X”

4)- Line 183: Possible bad formatting: “c” - It does not appear in Fig. 1 either; “y” “y” “z” “z”

5)- Fig. 1:

- shows g_s outputs y’, but in text you say g_s outputs x’

- h_a, h_s are not mentioned in text

- it’s not clear what’s the contribution of h_a, h_s to y’

6)- Lines 317-318: “a Attention” “an Attention”

7)- Line 347: “y” “y”

8)- Line 366: “has become” “becomes”

9)- Section 2.3: at what stage does uniform scalar quantization occur?

10)- Fig. 6: are ga, gs, ha, hs the same as g_a, g_s, h_a, h_s? If so, please use the same notation for consistency

11)- Line 420: “is adopt” “is adopted”

12)- Line 433: “Si” “Si”

13)- Line 526: you call it “compression ratio”, but what you show in Table 1 is actually (1 – 1 / CR) * 100%.

Reply 3.1: Thank you for your comments. Based on your comments, Based on your comments, we have made the following revisions.

1): In original Line 12, "bringing" is revised to "brings".

2): We have replaced reference [3] with a new related work.

3)-5): We have further refined Fig. 1 by explicitly labeling y, y', , z and z' in the corresponding positions of the compression framework. We have also clarified that the quantizer corresponds to the "Q" part of the "Q&CoDec" modules in Fig. 1. In addition, we added a detailed explanation of h_s and h_a. Specifically, h_a is the hyper-analysis transform that extracts the hyper-latent variable z from y, while h_s is the hyper-synthesis transform that estimates the probability distribution parameters of y' based on z'. These two modules constitute the auxiliary hyper-prior branch for entropy modeling rather than the main encoder and decoder. The detailed revisions can be found in the first paragraph after Fig. 1.

6): In original Lines 317-318, “a Attention” is revised to “an Attention”.

7): In original Line 347, “y” is revised as “y”.

8): In original Line 366, “has become” is revised to “becomes”.

9): In Section 2.3, the proposed uniform scalar quantization is used in the latent quantization stage, specifically in both the main latent representation y generated by g_a and the hyper-latent representation z generated by h_a. We have added the explanation in the paragraph before Equation (2).

10) : Thank you for pointing this out. The symbols "ga, gs, ha, hs" in fig. 6 have the same meanings as g_a, g_s, h_a and h_s. To avoid ambiguity, we have revised Fig. 6 and unified all symbols as g_a, g_s, h_a and h_s.

11): In original Line 420, “is adopt” is revised as “is adopted”.

12): In original Line 433, “Si” is revised as “Si”.

13): Thank you for this careful comment. We agree that the CR values in Table 1 do not correspond to the conventional compression ratio So/Sc. Instead, they represent the percentage of data volume reduced after compression, calculated as CR(%)=(1-Sc/So)* 100%. Therefore, we have revised the text before Table 1 by replacing "compression ratio" with "compression rate". We have also clarified the calculation formula and modified the table header accordingly to avoid confusion. The detailed revisions can be found in the paragraph before Table 1.

Attachment Submitted filename: Response to Reviewers.doc

# Peer review: Decision Letter 1

**DOI:** 10.1371/journal.pone.0355234.r003

**Authors:** Hashmat Fida

16 Jul 2026

PONE-D-26-16540R1

An End-to-End Deep Learning Image Compression Method for Satelllite Images based on Entropy Model

PLOS One

Dear Dr. Xu,

Thank you for submitting your manuscript to PLOS ONE. After careful consideration, we feel that it has merit but does not fully meet PLOS ONE’s publication criteria as it currently stands. Therefore, we invite you to submit a revised version of the manuscript that addresses the points raised during the review process.

Please submit your revised manuscript by Aug 29 2026 11:59PM. If you will need more time than this to complete your revisions, please reply to this message or contact the journal office at plosone@plos.org. When you're ready to submit your revision, log on to https://www.editorialmanager.com/pone/ and select the 'Submissions Needing Revision' folder to locate your manuscript file.

Please include the following items when submitting your revised manuscript:

- A letter that responds to each point raised by the academic editor and reviewer(s). You should upload this letter as a separate file labeled 'Response to Reviewers'.
- A marked-up copy of your manuscript that highlights changes made to the original version. You should upload this as a separate file labeled 'Revised Manuscript with Track Changes'.
- An unmarked version of your revised paper without tracked changes. You should upload this as a separate file labeled 'Manuscript'.

If you would like to make changes to your financial disclosure, please include your updated statement in your cover letter. Guidelines for resubmitting your figure files are available below the reviewer comments at the end of this letter.

If applicable, we recommend that you deposit your laboratory protocols in protocols.io to enhance the reproducibility of your results. Protocols.io assigns your protocol its own identifier (DOI) so that it can be cited independently in the future. For instructions see: https://journals.plos.org/plosone/s/submission-guidelines#loc-laboratory-protocols. Additionally, PLOS ONE offers an option for publishing peer-reviewed Lab Protocol articles, which describe protocols hosted on protocols.io. Read more information on sharing protocols at https://plos.org/protocols?utm_medium=editorial-email&utm_source=authorletters&utm_campaign=protocols.

As the corresponding author, your ORCID iD is verified in the submission system and will appear in the published article. PLOS supports the use of ORCID, and we encourage all coauthors to register for an ORCID iD and use it as well. Please encourage your coauthors to verify their ORCID iD within the submission system before final acceptance, as unverified ORCID iDs will not appear in the published article. *Only*  the individual author can complete the verification step; PLOS staff *cannot*  verify ORCID iDs on behalf of authors.

We look forward to receiving your revised manuscript.

Kind regards,

Dr. Hashmat Fida

Guest Editor

PLOS ONE

Journal Requirements:

If the reviewer comments include a recommendation to cite specific previously published works, please review and evaluate these publications to determine whether they are relevant and should be cited. There is no requirement to cite these works unless the editor has indicated otherwise.

Please review your reference list to ensure that it is complete and correct. If you have cited papers that have been retracted, please include the rationale for doing so in the manuscript text, or remove these references and replace them with relevant current references. Any changes to the reference list should be mentioned in the rebuttal letter that accompanies your revised manuscript. If you need to cite a retracted article, indicate the article’s retracted status in the References list and also include a citation and full reference for the retraction notice.

**Additional Editor Comments:**

After careful evaluation of the revised manuscript and the reviewers' comments, I am pleased to note that the manuscript has improved substantially. Reviewer #1 acknowledges that the majority of the previous concerns have been satisfactorily addressed. However, both reviewers have identified a few remaining issues that should be resolved before the manuscript can be considered for acceptance.

Accordingly, I invite you to submit a **minor revision**  addressing the following points:

Reviewer #1

- Confirm that the same randomly selected images from the VIIRS dataset were used across all compared methods to ensure a fair comparison.
- Include a brief summary of the comparative performance with existing methods in the Abstract.
- Revise the caption and the corresponding in-text reference for Table 3 to clarify that it summarizes comparisons with benchmark methods rather than benchmark datasets.
- Correct the language issues identified in Lines 598, 618, and 732, along with any remaining grammatical or typographical errors throughout the manuscript.

Reviewer #2

- Add a dedicated novelty comparison table that clearly distinguishes the proposed method from recent learned image compression approaches, including Ballé, Minnen, Cheng, ELIC, and Transformer-based methods.
- Include comparisons with relevant state-of-the-art learned image compression methods or provide a clear justification if such comparisons cannot be performed.
- Provide a component-wise ablation study demonstrating the contribution of each major module in the proposed framework. If an ablation study is not feasible, please explain the reason in the manuscript.
- Explicitly report the batch size and any other important training parameters used during model development to improve reproducibility.

Please provide a detailed **Response to Reviewers**  explaining how each comment has been addressed. Along with your response, please submit:

- A marked-up manuscript with all revisions highlighted (Track Changes).
- A clean version of the revised manuscript without tracked changes.

We appreciate your efforts in improving the manuscript and look forward to receiving your revised submission.

[Note: HTML markup is below. Please do not edit.]

Reviewers' comments:

Reviewer's Responses to Questions

**Comments to the Author**

1. If the authors have adequately addressed your comments raised in a previous round of review and you feel that this manuscript is now acceptable for publication, you may indicate that here to bypass the “Comments to the Author” section, enter your conflict of interest statement in the “Confidential to Editor” section, and submit your "Accept" recommendation.

Reviewer #1: All comments have been addressed

Reviewer #2: (No Response)

**********

2. Is the manuscript technically sound, and do the data support the conclusions?

The manuscript must describe a technically sound piece of scientific research with data that supports the conclusions. Experiments must have been conducted rigorously, with appropriate controls, replication, and sample sizes. The conclusions must be drawn appropriately based on the data presented.

Reviewer #1: Yes

Reviewer #2: Yes

**********

3. Has the statistical analysis been performed appropriately and rigorously?

Reviewer #1: Yes

Reviewer #2: Yes

**********

4. Have the authors made all data underlying the findings in their manuscript fully available?

The PLOS Data policy requires authors to make all data underlying the findings described in their manuscript fully available without restriction, with rare exception (please refer to the Data Availability Statement in the manuscript PDF file). The data should be provided as part of the manuscript or its supporting information, or deposited to a public repository. For example, in addition to summary statistics, the data points behind means, medians and variance measures should be available. If there are restrictions on publicly sharing data—e.g. participant privacy or use of data from a third party—those must be specified.

Reviewer #1: Yes

Reviewer #2: Yes

**********

5. Is the manuscript presented in an intelligible fashion and written in standard English?

PLOS ONE does not copyedit accepted manuscripts, so the language in submitted articles must be clear, correct, and unambiguous. Any typographical or grammatical errors should be corrected at revision, so please note any specific errors here.

Reviewer #1: No

Reviewer #2: Yes

**********

6. Review Comments to the Author

Please use the space provided to explain your answers to the questions above. You may also include additional comments for the author, including concerns about dual publication, research ethics, or publication ethics. (Please upload your review as an attachment if it exceeds 20,000 characters)

Reviewer #1: Round 2 review: 10.07.26

The authors have sincerely incorporated the suggestions given in my previous round of review. Compared the proposed work with other state of the art methods and included the suggested performance metrics. The new Table 3 states the same.

However, the authors have to confirm that the same set of randomly chosen images from the VIIRS data set is used with all the models for the comparison.

Minor comments:

1. A summary of the comparative performance with existing methods, should also be included in the abstract.

2. Line no. 578, the caption of Table 3 and its in-text reference needs to be changed. The table is a summary of the comparative performance metrics of the proposed work with the other “benchmarking methods” and “not benchmarking data sets”..

3. Line No. 598 --- sentence formation has to be corrected – “The processing process is as follows----”

4. Line No..618 ---sentence formation is very lucid –” Therefore, how to achieve direct and efficient compression….”

5. Line No.732 .. correct the word “vailable…”

Reviewer #2: The authors rewrote the novelty statement and clarified that their contribution is application-driven rather than claiming a fundamentally new architecture. However, they did not clearly distinguish their method from Ballé, Minnen, Cheng, or other state-of-the-art methods through a dedicated novelty comparison table.

The reviewer explicitly requested a novelty comparison table. No such table has been added.

No comparisons with modern learned image compression methods (Ballé, Cheng, ELIC, Transformer-based methods) have been added.

The manuscript discusses architectural components, but there is no proper component-wise ablation study demonstrating the contribution of each module independently.

Batch size is not explicitly reported.

**********

7. PLOS authors have the option to publish the peer review history of their article (what does this mean?). If published, this will include your full peer review and any attached files.

If you choose “no”, your identity will remain anonymous but your review may still be made public.

**Do you want your identity to be public for this peer review?**  For information about this choice, including consent withdrawal, please see our Privacy Policy.

Reviewer #1: No

Reviewer #2: No

**********

[NOTE: If reviewer comments were submitted as an attachment file, they will be attached to this email and accessible via the submission site. Please log into your account, locate the manuscript record, and check for the action link "View Attachments". If this link does not appear, there are no attachment files.]

To ensure your figures meet our technical requirements, please review our figure guidelines: https://journals.plos.org/plosone/s/figures

You may also use PLOS’s free figure tool, NAAS, to help you prepare publication quality figures: https://journals.plos.org/plosone/s/figures#loc-tools-for-figure-preparation.

NAAS will assess whether your figures meet our technical requirements by comparing each figure against our figure specifications.

Attachment Submitted filename: PLOS_One_round2_review.docx

# Author comment: Author response to Decision Letter 2

**DOI:** 10.1371/journal.pone.0355234.r004

16 Jul 2026

Dear Editor Hashmat Fida,

First of all, we would like to thank you very much for your time and effort in processing our paper entitled An end-to-end Deep Learning Image Compression Method based on Entropy Model (PONE-D-26-16540). We would also like to thank the reviewers for their valuable comments and constructive suggestions that help us improve the paper significantly.

As suggested by you, we have thoroughly checked our revised manuscript and made all the necessary changes. Thus we believe the quality and readability of the revised manuscript would be better.

Sincerely,

Heyang Xu

We once again thank the reviewers for their valuable comments. We have revised our paper to thoroughly incorporate the reviewers’ comments. In the following we respond to the comments of the reviewers one by one:

Reviewer #1

1. Confirm that the same randomly selected images from the VIIRS dataset were used across all compared methods to ensure a fair comparison.

Reply 1.1: Thank you for your comments. Based on your suggestions, we have conducted more experimental studies on the metrics of MSE, PSNR, bpp and SSIM and the results are listed in Table 3. The details can be found in Table 3 and the paragraphs before and after Table 3 in Section 4.3.

2. Include a brief summary of the comparative performance with existing methods in the Abstract.

Reply 1.2: Thank you for your comments. Based on your suggestions, we have added a brief summary of the comparative performance with existing methods in the Abstract. The added summary is “....the proposed method achieves 0.51±0.04 bpp, 38.39±0.72 dB PSNR and 0.973±0.007 SSIM. Relative to ELIC and the Transformer-CNN baseline, it reduced bpp by 13.6% and 10.5% and improved PSNR by 0.78 dB and 0.61 dB, respectively.”

3.Revise the caption and the corresponding in-text reference for Table 3 to clarify that it summarizes comparisons with benchmark methods rather than benchmark datasets.

Reply 1.3: Thank you for your comments. Based on your comments, we have revised the caption of Table 3 and the corresponding in-text reference. The detailed revisions are as follows:

First, the caption of Table 3 is revised to “Table 3. The results of the performance metrics obtained by compared methods.”

Second, the corresponding in-text reference for Table 3 is revised to “The results of the comparative performance metrics of the proposed work with the other benchmark methods are list in Table 3.”

4. Correct the language issues identified in Lines 598, 618, and 732, along with any remaining grammatical or typographical errors throughout the manuscript.

Reply 1.4: Thank you for your comments. Based on your suggestions, we have carefully proofread and made the following revisions.

1): The sentence “The processing process is as follows----” is revised to “The processing steps are as follows-----.”

2): The last sentence in the first paragraph of Section “5. Conclusions” is revised to “As a natural extension of this study, the exploration of direct, high-efficiency compression techniques for large-scale satellite image data will be carried out in our future work.”

3): The word “vailable” in Reference [37] is revised to “available”.

4): The word “Satelllite” in the title of the paper is revised to “Satellite”.

And some other changes are made which are not listed here for the sake of brevity.

Reviewer #2

1.Add a dedicated novelty comparison table that clearly distinguishes the proposed method from recent learned image compression approaches, including Ballé, Minnen, Cheng, ELIC, and Transformer-based methods.

Reply 2.1: Thank you for your comments. Based on your comments, we have reorganized Table 1.

2. Include comparisons with relevant state-of-the-art learned image compression methods or provide a clear justification if such comparisons cannot be performed.

Reply 2.2: Thank you for your comments. Based on your comments, the comparative evaluation has been broadened to include representative modern learned-compression baselines, including Ballé scale hyperprior, Minnen joint autoregressive-hyperprior, Cheng Gaussian-mixture-attention, ELIC and a mixed Transformer-CNN method. To avoid confusion, we have explicitly pointed this out in the paragraph before Table 3. Also, in the discussion paragraph after Table 3, we acknowledge that the margins over recent learned methods are smaller than those over classical codecs.

3.Provide a component-wise ablation study demonstrating the contribution of each major module in the proposed framework. If an ablation study is not feasible, please explain the reason in the manuscript.

Reply 2.3: Thank you for your comments. Based on your comments, we have added a component-wise leave-one-out ablation section to isolate the effects of the residual bottleneck blocks, simplified attention modules, Gaussian-mixture entropy model and checkerboard context. The detailed revisions can be in Section “4. 4 Component-Wise Ablation Study.”

4. Explicitly report the batch size and any other important training parameters used during model development to improve reproducibility.

Reply 2.4: Thank you for your comments. Based on your comments, we have expanded the experimental setup to include the batch size, optimizer parameters, learning-rate schedule, number of epochs, rate-distortion weights, software versions, hardware, test-set size and so on. The detailed revisions can be found in Section “4.2 Experimental Setup.”

We again thank the reviewers for their constructive comments, which have substantially improved the clarity and technical completeness of the manuscript.

Attachment Submitted filename: Response_to_Reviewers_auresp_2.doc

# Editor report: Decision Letter 2

**DOI:** 10.1371/journal.pone.0355234.r005

**Authors:** Hashmat Fida

19 Jul 2026

**Manuscript Title:**  *An End-to-End Deep Learning Image Compression Method for Satellite Images Based on Entropy Model*

**Manuscript ID:**  **PONE-D-26-16540R2**

Dear Dr. Xu,

We are pleased to inform you that your manuscript has been judged **scientifically suitable for publication**  in **PLOS ONE** . The manuscript will be **formally accepted**  once all outstanding technical and production requirements have been successfully completed.

Within the next week, you will receive an email outlining any required technical amendments. After these requirements have been addressed and verified, you will receive a formal acceptance letter, and your manuscript will proceed to the publication stage.

An invoice will be generated once your article is formally accepted. Please note that if your institution participates in a PLOS publishing partnership and your article meets the applicable eligibility criteria, all or part of your publication charges may be covered. To help ensure accurate processing, please verify that your contact and institutional information are up to date in Editorial Manager by selecting **"Update My Information."**  For any billing-related inquiries, please contact the PLOS Billing Support team.

If your institution has a press office, we encourage you to notify them about your forthcoming publication to maximize its visibility and impact. Should they plan to prepare press materials, please inform the PLOS press team as soon as possible, and no later than **48 hours after receiving your formal acceptance** . Please note that your manuscript will remain under strict press embargo until **2:00 PM Eastern Time**  on the date of publication.

Congratulations on your successful submission, and thank you for choosing **PLOS ONE**  for the publication of your work.

Kind regards,

**Dr. Hashmat Fida**

Guest Editor

**PLOS ONE**

**********

<h3>Additional Editor Comments (Optional)</h3>

Dear Authors,

Thank you for your careful and comprehensive revision of the manuscript. The revised version has satisfactorily addressed the concerns raised during the previous round of peer review, particularly regarding the comparative evaluation, clarification of the manuscript's novelty, experimental design, reproducibility, and the overall quality of presentation. The manuscript has been substantially strengthened and now demonstrates improved technical rigor, clarity, and completeness.

I appreciate the effort invested in responding to the reviewers' comments in a thorough and constructive manner. I have no further major comments and believe the manuscript is suitable for publication.

I congratulate the authors and wish you continued success in your future research endeavors.

**********

<h3>Reviewers' Comments</h3>

No additional comments. The concerns raised during the previous review rounds have been satisfactorily addressed. The manuscript is recommended for publication.

# Editor report: Acceptance letter

**DOI:** 10.1371/journal.pone.0355234.r006

**Authors:** Hashmat Fida

PONE-D-26-16540R2

PLOS One

Dear Dr. Xu,

I'm pleased to inform you that your manuscript has been deemed suitable for publication in PLOS One. Congratulations! Your manuscript is now being handed over to our production team.

At this stage, our production department will prepare your paper for publication. This includes ensuring the following:

* All references, tables, and figures are properly cited

* All relevant supporting information is included in the manuscript submission,

* There are no issues that prevent the paper from being properly typeset

You will receive further instructions from the production team, including instructions on how to review your proof when it is ready. Please keep in mind that we are working through a large volume of accepted articles, so please give us a few days to review your paper and let you know the next and final steps.

Lastly, if your institution or institutions have a press office, please let them know about your upcoming paper now to help maximize its impact. If they'll be preparing press materials, please inform our press team within the next 48 hours. Your manuscript will remain under strict press embargo until 2 pm Eastern Time on the date of publication. For more information, please contact onepress@plos.org.

You will receive an invoice from PLOS for your publication fee after your manuscript has reached the completed accept phase. If you receive an email requesting payment before acceptance or for any other service, this may be a phishing scheme. Learn how to identify phishing emails and protect your accounts at https://explore.plos.org/phishing.

If we can help with anything else, please email us at customercare@plos.org.

Thank you for submitting your work to PLOS ONE and supporting open access.

Kind regards,

PLOS ONE Editorial Office Staff

on behalf of

Dr. Hashmat Fida

Guest Editor

PLOS One
